# Floral plasticity: Herbivore‐species‐specific‐induced changes in flower traits with contrasting effects on pollinator visitation

**DOI:** 10.1111/pce.13520

**Published:** 2019-03-08

**Authors:** Quint Rusman, Erik H. Poelman, Farzana Nowrin, Gerrit Polder, Dani Lucas‐Barbosa

**Affiliations:** ^1^ Laboratory of Entomology Wageningen University Wageningen The Netherlands; ^2^ Greenhouse Horticulture Wageningen University, Wageningen The Netherlands

**Keywords:** *Brassica nigra* (black mustard), flower colour, flower morphology, flower rewards, flower volatiles, herbivore‐induced plant responses, phenotypic plasticity, plant defence, plant‐mediated interactions, specificity

## Abstract

Plant phenotypic plasticity in response to antagonists can affect other community members such as mutualists, conferring potential ecological costs associated with inducible plant defence. For flowering plants, induction of defences to deal with herbivores can lead to disruption of plant–pollinator interactions. Current knowledge on the full extent of herbivore‐induced changes in flower traits is limited, and we know little about specificity of induction of flower traits and specificity of effect on flower visitors. We exposed flowering *Brassica nigra* plants to six insect herbivore species and recorded changes in flower traits (flower abundance, morphology, colour, volatile emission, nectar quantity, and pollen quantity and size) and the behaviour of two pollinating insects. Our results show that herbivory can affect multiple flower traits and pollinator behaviour. Most plastic floral traits were flower morphology, colour, the composition of the volatile blend, and nectar production. Herbivore‐induced changes in flower traits resulted in positive, negative, or neutral effects on pollinator behaviour. Effects on flower traits and pollinator behaviour were herbivore species‐specific. Flowers show extensive plasticity in response to antagonist herbivores, with contrasting effects on mutualist pollinators. Antagonists can potentially act as agents of selection on flower traits and plant reproduction *via* plant‐mediated interactions with mutualists.

## Abstract 2

We show that plant interactions with pollinators may be easily disrupted or enhanced by herbivores due to extensive floral plasticity. Herbivore‐species‐specific changes in flower morphology, colour, volatile blend, and nectar and pollen production had contrasting effects on pollinator behaviour.

## 1 INTRODUCTION

Plants interact with an incredibly diverse community of plant mutualists and antagonists. Antagonists range from large mammals to tiny insects, and microscopic bacteria and viruses. Each of these attackers may differ in the mode of attack as well as the fitness costs associated with the attack. To successfully defend against this plethora of attackers, plants evolved various defensive strategies (Agrawal, 2011; Dicke & van Loon, 2014; Karban, 2011). These strategies include inducible defences that allow resistance to be fine‐tuned to the specific attacker and save metabolic costs of resistance in the absence of herbivores (Karban, 2011; Karban & Baldwin, 1997; Kessler, 2015). However, such phenotypic plasticity can affect many other interactions between the plant and its environment in addition to the target herbivore, with potential negative effects on plant fitness, imparting so‐called ecological costs (Heil, 2002; Poelman, 2015; Poelman & Kessler, 2016; Strauss, Rudgers, Lau, & Irwin, 2002). Ecological costs of phenotypic plasticity are most clearly revealed in flowering plants. The majority of flowering plants are involved in one or more intimate interactions with pollinators, and disruption of plant–pollinator interactions can be directly detrimental for plant fitness (Ollerton, Winfree, & Tarrant, 2011; Wilcock & Neiland, 2002). A number of studies have identified disruptions in plant–pollinator interactions due to plant responses to insect herbivores (Hoffmeister, Wittköpper, & Junker, 2016; Liao, Gituru, Guo, & Wang, 2013; Schiestl, Kirk, Bigler, Cozzolino, & Desurmont, 2014), with consequences for plant reproduction (Botto‐Mahan et al., 2011; Chautá, Whitehead, Amaya‐Márquez, & Poveda, 2017; Rusman, Lucas‐Barbosa, & Poelman, 2018).

Herbivore‐induced changes in pollinator visitation are mediated by plasticity in flower traits. Plants attract pollinators through various flower traits: flower abundance, size, morphology, colour, volatiles, and rewards (nectar and pollen; Akter, Biella, & Klecka, 2017; Junker & Parachnowitsch, 2015). Flower traits are highly plastic and change readily in response to environmental factors, such as herbivory (Lucas‐Barbosa, van Loon, & Dicke, 2011; Strauss, 1997). Changes in response to herbivory include most flower traits involved in pollinator attraction (Bruinsma et al., 2014; Cozzolino et al., 2015; Hoffmeister et al., 2016; Lucas‐Barbosa et al., 2016). Herbivore‐induced changes in floral traits may vary considerably depending on herbivore species (Pareja et al., 2012; Rusman et al., 2018). In our previous work, we identified that herbivore–pollinator interactions may depend on feeding guild and site of the herbivore as well, but we did not characterize which flower traits were responsive to herbivory (Rusman et al., 2018). Thus, limited knowledge is available of the full extent to which different flower traits are affected by herbivore induction, the specificity of induction of flower traits, and specificity of effects on flower visitors. We think that knowledge on the specificity of herbivore‐induced plant responses provides an opportunity to explore the extent to which plasticity in floral traits supports or leads to disruption of plant–pollinator interactions.

We expect flower plasticity to follow patterns of specificity in inducible defences known for foliar plant responses. Specificity in inducible defences is to some extent mediated by phytohormones, which are involved in defence as well as reproduction. Chewing herbivores, for instance, mainly induce the jasmonic acid (JA) pathway, whereas sap‐feeding herbivores usually suppress JA and/or induce the salicylic acid (SA) pathway (Ali & Agrawal, 2012; Erb, Meldau, & Howe, 2012; Thaler, Humphrey, & Whiteman, 2012). Root‐feeding herbivores induce the JA pathway, but the phytohormonal network seems different belowground compared with aboveground, resulting in different plant responses (Johnson, Erb, & Hartley, 2016). Flower traits are also regulated by these phytohormones. For example, SA regulates flowering time (Martínez, Pons, Prats, & León, 2004) and is involved in flower formation (Rivas‐San Vicente & Plasencia, 2011) and flower thermogenesis (Raskin, Ehmann, Melander, & Meeuse, 1987). Likewise, JA is involved in general developmental processes of flowering (Yuan & Zhang, 2015) and regulates the expression of various flower traits (Avanci, Luche, Goldman, & Goldman, 2010; Brioudes et al., 2009; Muhlemann, Klempien, & Dudareva, 2014; Radhika, Kost, Boland, & Heil, 2010). Moreover, flower and defence traits are linked via shared genetic or biochemical pathways, via shared resources (Jacobsen & Raguso, 2018), or via functional responses, where flower traits are involved in defence as well. Flowering plants use floral volatiles to attract pollinators but also natural enemies of herbivores (Lucas‐Barbosa et al., 2014; Schiestl et al., 2014), and although pigments colour the flower, they are also toxic for herbivores (Gronquist et al., 2001). Because of the multiple links between flower and defensive traits, we expect similarities in specificity of herbivore induction.

In this study, we tested whether responses of the annual *Brassica nigra* to feeding by various herbivores affect multiple flower traits and whether herbivore‐induced changes in flower traits affect pollinator behaviour. We specifically studied how herbivore‐induced plant responses affect (a) flower abundance, size, and morphology; (b) flower chemistry, such as colour and odours; (c) flower rewards, including nectar and pollen quantity and pollen size; and (d) the behaviour of two pollinating insects, the butterfly *Pieris brassicae* and the syrphid fly *Episyrphus balteatus*. We hypothesized that herbivores from similar feeding guilds/sites induce more similar changes in flower traits than do herbivores from different feeding guilds/sites. This is predicted by specificity of elicitation of phytohormones involved in defence regulation (Ali & Agrawal, 2012; Johnson et al., 2016; Thaler et al., 2012) and different effects of plant responses to herbivores from various feeding guilds and feeding sites on pollinators (Rusman et al., 2018).

## 2 MATERIALS AND METHODS

### 2.1 Plants and insects

Black mustard (*B. nigra* L., Accession CGN06619) seeds were obtained from field open‐pollinated plants and originated from the Centre for Genetic Resources (Wageningen, the Netherlands). Seeds were germinated in trays, and 1‐week‐old plants were transplanted and cultivated in pots (∅ 17 cm, 2 L) filled with potting soil (Lentse potgrond) and sand in a 1:1 volume ratio under greenhouse conditions (23 ± 2°C, 50–70% relative humidity [RH], L16:D8). Once plants started flowering (5 or 6 weeks old), they were used for the experiments.

We exposed plants to six herbivore species divided over three groups, here termed “herbivore functional groups” (HFGs): chewing herbivores (larvae of *Athalia rosae* L., *Plutella xylostella* L., and *P. brassicae* L.), sap‐feeding herbivores (adults of *Brevicoryne brassicae* L. and *Lipaphis erysimi* (Kaltenbach)) and root herbivores (larvae of *Delia radicum* L.). All herbivores are specialists on Brassicaceae. The sawfly *A. rosae* originated from surroundings of Würzburg (Bavaria, Germany). The larvae were reared on *Raphanus sativus* under greenhouse conditions (22 ± 1°C, 50–70% RH, L16:D8). The caterpillars *P. xylostella* and *P. brassicae* and aphids *B. brassicae* and *L. erysimi* originated from the surroundings of Wageningen (the Netherlands), and they are routinely reared in the Laboratory of Entomology (Wageningen University) under greenhouse conditions (22 ± 1°C, 50–70% RH, L16:D8). *Plutella xylostella*, *P. brassicae*, and *B. brassicae* were reared on Brussels sprouts plants (*Brassica oleracea* var. *gemmifera* cv. Cyrus); *L. erysimi* was reared on *R. sativus* plants. The cabbage root fly *D. radicum* originated from Saint‐Méloir‐des‐Ondes (Brittany, France). Larvae were reared on turnips (*Brassica rapa*) or rutabaga (*Brassica napus*) in a climate cabinet (22 ± 1°C, 50–70% RH, L16:D8). We used two different pollinator species for our experiments: the butterfly *P. brassicae* and the syrphid fly *E. balteatus* (De Geer). Butterflies mainly feed on nectar, and not pollen, while feeding on *B. nigra* plants. Compared with other pollinators, *P. brassicae* has a low visitation frequency in the field (Lucas‐Barbosa, van Loon, Gols, Beek, & Dicke, 2013; Rusman et al., 2018) but may nonetheless be important for long‐distance pollen dispersal (Courtney, Hill, & Westerman, 1982). Male and female *P. brassicae* butterflies were placed together to mate at 1–3 days after eclosing. After mating, females were provided with a 10% honey solution until being used in our behavioural experiments. *E. balteatus* pupae were obtained from Koppert Biological Systems (Berkel en Rodenrijs, the Netherlands). Adult syrphid flies were provided with sugar, pollen, water, and a Brussels sprouts plant infested with *B. brassicae* aphids, which is known to promote the development of the female reproductive system. Syrphid flies can feed on both nectar and pollen but mainly feed on pollen of *B. nigra*. *E. balteatus* is a common flower visitor and efficient pollinator of Brassicaceae (Jauker & Wolters, 2008). For both pollinators, we only used females for the behaviour experiment.

### 2.2 Plant treatments

We infested flowering *B. nigra* plants, 1 to 3 days from the start of flowering, by placing 10 first‐instar sawfly larvae or caterpillars, or 20 adult aphids on the two lowest bracts (5/10 per bract), or 10 first instar larvae of the root herbivore *D. radicum* at the base of the stem. Aboveground herbivores were not constrained to the bracts and free to move to their preferred feeding sites. Herbivore infestation densities were based on field observations and equalized for HFGs. After 7 days of herbivore infestation, plants were used in the experiments (Chrétien et al., 2018), and various flower traits (abundance, size, morphology, colour, volatiles, nectar production, and pollen quantity and size) and pollinator behaviour were assessed. For volatile collection, insects were removed prior to the experiment. For other flower traits, we always sampled undamaged flowers and inflorescences. After experimental use, we recorded the number and instar of all aboveground herbivore species, and root samples were taken for belowground herbivore‐infested and uninfested control plants to assess the actual damage caused by *D. radicum*. Control plants were kept uninfested for 7 days.

### 2.3 Effect of herbivore infestation on flower size, morphology, and colour

To investigate if flower size, morphology, and colour were influenced by herbivore infestation, we measured the size, several morphological features, and the diffuse colour reflectance of flowers of plants infested with one of five herbivore species (*A. rosae*, *P. xylostella*, *B. brassicae*, *L. erysimi*, and *D. radicum*) and uninfested plants. Whole flowers (single open uninfested flowers) were mounted on a platform made of cork (diameter 2.3 cm), located under a multispectral camera (Pixelteq SpectroCam; resolution 1.3 Mp; lens = Carl Zeiss 2.8/25 ZF‐IR) at 11.4 cm. The multispectral camera was equipped with eight filters (Table S1). Halogen light (KL1500 fibre optic light source, Schott, Mainz, Germany) was provided from the top, next to the camera under a slight angle, from two sides. After a top‐view picture of the whole flower was taken, the petals were separated from the rest of the flower, and a top‐view picture was taken from the four petals together. After multispectral image capture, the cork platform containing the petals was transferred to a spectroscopy set‐up. For each petal, we measured the top (centred, 0.5 mm below the top edge of the petal) and the base (centred, 0.5 mm above the point where the petal narrows and bend downwards) with a spectrometer (SD2000, Ocean Optics, Largo, FL) using a fibre optic reflection probe and a deuterium‐halogen light source (DH2000‐FHS, Ocean Optics, Largo, FL) under a 162.5° angle at 1–2 mm. The spectrometer was calibrated using white (WS‐2, TOP Sensory Systems) and black (by covering the input fibre) as references. The diffuse reflection spectrum from 300 to 700 nm and two regions of interest, the yellow/orange region (570–650 nm) and the UV region (310–370 nm), were taken as read‐out. Six flowers of the final inflorescence of the top two flowering branches (three flowers per inflorescence) were measured from each plant, and flowers of six to eight plants per treatment. We processed whole flower and petal images by creating a segmentation pipeline: A supervised Gaussian classification model was built using three images of flowers and petals for separating flower and background. All other images were segmented by this trained classification model. This was done in MATLAB (Version R2017b) with the perClass toolbox (perClass Enterprise 5.2, PR Sys Design, Delft, the Netherlands). We inspected each image after automated segmentation and manually corrected the segmentation where needed in Paint (Version 6.1). Following segmentation, 42–48 flower images per treatment were analysed for surface area, surface area perimeter, convex area, convex area perimeter, length and width of fitting ellipse (major and minor chord length), and eccentricity (shape of fitting ellipse). With these measurements, we calculated the aspect ratio (major/minor chord length), solidity (surface area/convex area), and convexity (convex area perimeter/surface area perimeter). We analysed 164–196 petal images per treatment for surface area, length and width of fitting ellipse, diameter, and eccentricity of individual petals.

### 2.4 Effect of herbivore infestation on plant volatile emission

To investigate if volatile emission of flowering plants was influenced by herbivore infestation, we collected volatiles from plants infested with one of five herbivore species (*A. rosae*, *P. xylostella*, *B. brassicae*, *L. erysimi*, and *D. radicum*) and uninfested plants. We collected volatiles of the aboveground plant parts, both leaves and flowers, because it was impossible to exclude all leaves from the inflorescences. Although we cannot separate volatiles from leaves and flowers, volatiles of leaves comprise only 2% of the total volatile emission of flowering *B. nigra* plants and did not respond to herbivory in a previous study (Bruinsma et al., 2014). Thus, herbivore‐induced changes in plant volatile emission of flowering plants are most likely due to changes in floral volatiles. We enclosed the aboveground plant parts in an oven bag (Toppits® Bratschlauch, polyester, 32 cm × 32 cm × 70 cm; Toppits, Minden, Germany). During collection, synthetic air from a gas cylinder was flushed through the bag at a flow rate of 300 ml min^−1^ by inserting a Teflon tube through an opening in the upper part of the bag, and air was sucked out (224‐PCMTX8, air‐sampling pump Deluxe, Dorset, UK; equipped with an inlet protection filter) at a flow rate of 200 ml min^−1^ through a second Teflon tube at the opening of each bag, and volatiles were collected in a metal tube filled with Tenax‐TA for 1.5 hr. Collections were done in a greenhouse compartment (25 ± 1°C, 50–70% RH, L16:D8) between 12 pm and 2 pm, and volatiles of six to eight plants were collected for each treatment. After collection, headspace samples were analysed using a gas chromatograph with a thermodesorption unit and coupled to a mass spectrometer (Thermo Fisher Scientific, Waltham, MA). Plant volatiles were desorbed from the Tenax using a thermodesorption unit (Ultra 50:50, Markes, Llantrisant, UK) that heated the sample from 25°C to 250°C (5‐min hold) in splitless mode at a rate of 60°C min^−1^. The released compounds were focused in a cold trap (internal diameter [ID] 1.80 mm) at 0°C, filled with Tenax and charcoal. The volatiles were transferred in splitless mode to the analytical column (30 m × 0.25 mm ID, 1‐μm film thickness, DB‐5, Phenomenex, Torrence, CA) by flash heating the cold trap at 40°C s^−1^ to 280°C (hold 10 min) and was held for 4 min at constant flow of 1 ml min^−1^. The temperature programme of the oven started at 40°C, and it immediately rose at 5°C min^−1^ to 280°C (4‐min hold) with constant flow of 1 ml min^−1^. An electron impact ionization at 70 eV was used to ionize the column effluent. Mass scanning was carried out from *m*/*z* 35 to 300 with 4.70 scans s^−1^. Compounds were putatively identified by comparing the mass spectra with the mass spectra of Wiley libraries and the Wageningen Mass Spectral Database of Natural Products. Identified compounds were confirmed on the basis of retention index using the literature (Adams, 1995). The emission rates were only quantified for compounds that were detected in a minimum of 50% of the samples from one of the treatments, and peak area of individual compounds was divided by fresh plant biomass (grams). Total ion counts were obtained to generate values for peak area, and we used these values to calculate the total volatile emission of plants; a single ion was selected to generate values for peak area and used when analysing the volatile blend composition of *B. nigra* plants.

### 2.5 Effect of herbivore infestation on floral nectar and pollen

To investigate if floral nectar and pollen production were affected by herbivore infestation, we measured nectar and pollen quantity and pollen size of uninfested and herbivore‐infested plants. Nectar was collected from eight flowers between 9 a.m. and 10 a.m. in the morning by using a 2‐μl glass capillary (Microcaps®); three flowers of the final inflorescence of the two top flowering branches and two flowers of the final inflorescence of the third flowering branch from the top were selected, and all flowers were 2 days old. Nectar of 15 plants was collected for plants infested with one of five herbivore species (*A. rosae*, *P. brassicae*, *B. brassicae*, *L. erysimi*, and *D. radicum*) and uninfested plants. Pollen quantity and size was measured using a flow cytometer: Multisizer II Coulter Counter (Beckman Coulter (UK) Ltd., High Wycombe, UK). The six anthers of a flower were collected in a 2‐ml Eppendorf tube with 0.5 ml of soap water to prevent clumping of pollen grains. The anthers were crushed with a glass rod and then vortexed. Samples were then poured through a filter (MACS® SmartStainers, Miltenyi Biotec, Germany) with a pore size of 100 μm to get rid of debris. The filter was flushed with 6.5 ml of water to collect pollen grains stuck in the filter. We then added 13 ml of isotonic electrolyte Isoton (Isoton® II diluent, Beckman Coulter (UK) Ltd., High Wycombe, UK), and we homogenized the samples prior to measurements by whirling the container around. The flow cytometer measured the number and size of all particles in 1 ml of solution. To exclude debris (crushed anther tissue, etc.), we selected particles between 20 and 30 μm in our dataset on the basis of the particle size distribution of the samples. We multiplied the number of pollen grains in 1 ml by 20 to estimate the total number of pollen grains per flower. Pollen was collected from five flowers per plant, three flowers from the final inflorescence of the top flowering branch, and two flowers from the final inflorescence of the second flowering branch from the top. All flowers used were 2 days old. Pollen of 10 plants was collected for plants infested with one of three herbivore species (*P. brassicae*, *L. erysimi*, and *D. radicum*) and uninfested plants.

### 2.6 Effect of herbivore infestation on pollinator behaviour

To investigate if pollinator behaviour was influenced by plant infestation, we recorded the behaviour of two pollinators, the butterfly *P. brassicae* and the syrphid fly *E. balteatus*, in two choice situations. Individual pollinators were offered two plants: an uninfested plant and a plant infested with one of five herbivore species (*A. rosae*, *P. xylostella*, *B. brassicae*, *L. erysimi*, and *D. radicum*). A single butterfly or syrphid fly was released at a time, at 100 cm from the plants. Each pollinator was observed for 12 min, and we recorded the plant of first choice, and the visitation time and number of flowers visited for each of the two plants. First choice was defined as the plant first contacted by the insect, with either a leaf or flower. If the pollinator did not make a choice within 5 min, it was recorded under “no response,” and the observation was terminated. Observations were performed using a handheld computer (Psion Workabout Pro^tm^ 3, London, UK) programmed with The Observer XT software (Version 10, Noldus Information Technology, Wageningen, the Netherlands). Each pollinator was used only once. Butterflies were 3–10 days old, starved approximately 20 hr before the experiment, and provided with a Brussels sprout plant to lay eggs; and thus, in this way, we ensured that the observation time would be spent on feeding and not on oviposition. Syrphid flies were 5–15 days old, starved 4–8 hr before the experiment, and provided with a Brussels sprouts plant to lay eggs and some water to prevent dehydration. For each plant pair, 10–20 individuals per pollinator species were tested. If more than 10 individuals were nonresponsive, observations for that day were terminated and data were excluded from the dataset. Experiments were carried out in a flight chamber set‐up (gauze tent of 293 cm × 200 cm × 230 cm), in a greenhouse compartment (25 ± 1°C, 50–70% RH, L16:D8). For each plant treatment, seven to 10 plant pairs were tested. After experimental use, we recorded the number of flowers and inflorescences for each plant.

### 2.7 Statistical analysis

For count data such as the number of flowers, inflorescences, and pollen grains, we used generalized linear (mixed) models with a Poisson distribution and a log link function or negative binomial distribution with a log link function to correct for overdispersion. Herbivore species was included in the model as fixed factor. For post hoc analysis, we used Tukey's post hoc tests. Random factors were selected using a backwards approach; all random factors such as *block* (flowers and inflorescences) and *plant* (pollen grains) were initially added to the model and removed if they explained less than 5% of the variation or were statistically nonsignificant (*P* > 0.05). We used the lme4 (Bates, Maechler, Bolker, & Walker, 2015), multcomp (Hothorn, Bretz, & Westfall, 2008), and lmtest (Zeileis & Hothorn, 2002) packages for these analyses. For continuous data such as total volatile emission, emission of compound classes or individual compounds, relative reflection of yellow and UV, amount of nectar, and average pollen size, we used linear (mixed) models with a Gaussian distribution and identity link function or a Gamma distribution with a log link function if the data did not follow a normal distribution. The same fixed factors, random factor selection approach, and packages as for count data were used. Random factor used was *plant* (yellow, UV, and pollen size). For pollinator behaviour data (number of insects, flowers visited, time spent per plant, and flower), we used the proportion of the response variable between infested and uninfested plants. We used generalized linear mixed models (GLMMs) with a Poisson distribution and a log link function. The response variable was fitted to the intercept, and random factor used was *plant pair*.

We analysed reflectance spectra with principal component analysis, permutational multivariate analyses of variance (PERMANOVAs), and support vector machines (SVMs; Cortes & Vapnik, 1995). Principal component analysis was performed with MetaboAnalyst 4.0 with default settings (Xia, Sinelnikov, Han, & Wishart, 2015). For PERMANOVAs, the difference in reflectance spectra between the top and base parts of petals was analysed by including herbivore treatment, plant identity, flower identity, and petal part as fixed factors. The difference in reflectance spectra between herbivore treatments for the top and base parts of petals was analysed by including herbivore treatment, flower identity, and plant identity as fixed factors. We used 999 permutations for all PERMANOVAs. We used the vegan package for these analyses (Oksanen et al., 2007). For SVM, the reflectance spectra were divided into 64 features of 4–5 nm each. We trained the models with 60% of the data, used 20% for model optimization and cross‐validation, and used 20% for testing. For each model, we tested a range of cost values (2^−2:7^ with exponential steps of 1) and gamma values (from 0 to 1 with steps of 0.05) and selected the values that supported the best model. For gamma, values auto‐selected by the model always supported the best models compared with a range of test values selected by us (between 0 and 1 with steps of 0.05), and consequently, we used the values auto‐selected by the model, which were always <0.01. Accuracy was assessed with 10‐fold cross‐validation. Error rate was calculated from the confusion matrix by dividing the sum of the diagonals by the sum of the total. The result was subtracted from 1. We used the e1071 package for these analyses (Meyer, Dimitriadou, Hornik, Weingessel, & Leisch, 2017). For all reflectance spectrum analyses, we excluded wavelengths 421 to 498 from our dataset because of low light intensity in this area of the deuterium‐halogen light source.

We analysed volatile blend composition with cluster analysis. For cluster analysis, data were averaged per treatment, log transformed, and range scaled. Clustering was done using Euclidian distances as distance measure and Ward's clustering criterion as clustering method. Bootstrapping was done with 1,000 bootstrap replications. We used the pvclust package for these analyses (Suzuki & Shimodaira, 2006). Heat maps were produced with MetaboAnalyst 4.0 with default settings, except we did not use standardization (Xia et al., 2015).

We analysed pollen size distribution by comparing the pollen size distribution of uninfested and infested plants with a *χ* ^2^ test. We used the fifer package for these analyses (Fife, 2014). All analyses were carried out in R (Version 3.4.3 × 64, 2017, The R Foundation for Statistical Computing Platform).

## 3 RESULTS

Herbivore induction affected most flower traits and pollinator behaviour (Table 1). Changes in response to herbivory were observed for flower morphology, flower colour, the composition of the volatile blend, and nectar and pollen production of flowering *B. nigra*. Except for flower colour, specificity in induced traits and pollinator behaviour was herbivore species‐specific and beyond classifications such as HFGs. For example, both pollinators had opposite responses to plants infested with the two aphid species (Table 1). Moreover, herbivore‐induced changes were not restricted to specific herbivores or traits, and different herbivore species induced changes in various traits. Below, we provide details for the effects of plant responses to herbivory for each of the flower traits and the behaviour of the pollinators.

**Table 1:**
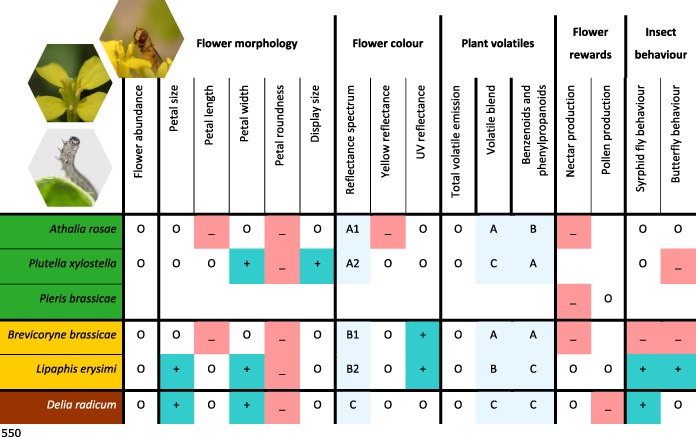
Various flower traits of *Brassica nigra* and visitation by two pollinators for plants infested with different herbivores

*Note*. Increase (+), decrease (−), or no effect (O) when compared with traits of uninfested plants. Letters for volatile blend and compound class are based on cluster analyses. Letters and numbers for reflectance spectrum are based on support vector machine models, where letters indicate differences between feeding guilds and numbers indicate differences within feeding guilds. No entry indicates traits were not measured for the respective herbivore. Photographs show *Athalia rosae* larva (bottom), flower of *B. nigra* (centre), and adult syrphid fly *Episyrphus balteatus* feeding on pollen of *B. nigra* (top right). Photograph credits: Jitte Groothuis and Quint Rusman.

### 3.1 Effect of herbivore infestation on flower abundance, size, and morphology

On average, plants had 364 flowers and 34 inflorescences 1 week after the start of flowering. Herbivory did not affect the number of inflorescences (Figure S1; GLMM: *χ* ^2^ = 2.20, *df* = 5, *P =* 0.821) nor the number of flowers (Figure S1; GLMM: *χ* ^2^ = 1.47, *df* = 5, *P =* 0.916). Flowers had an average display area of 1.0 cm^2^ and length and width of 1.2 × 1.0 cm. Herbivory affected the display size of flowers (Figure S2; generalized linear model [GLM]: *χ* ^2^ = 46.3, *df* = 5, *P* < 0.001), and flowers of plants infested with *P. xylostella* were 18% larger than flowers of uninfested plants (Tukey's post hoc tests, *P* < 0.001). Herbivory affected several shape characteristics of flowers (Figure S2), such as major chord length (GLM: *χ* ^2^ = 36.5, *df* = 5, *P* < 0.001), minor chord length (GLM: *χ* ^2^ = 25.3, *df* = 5, *P* < 0.001), aspect ratio (GLM: *χ* ^2^ = 11.7, *df* = 5, *P =* 0.039), solidity (GLM: *χ* ^2^ = 14.9, *df* = 5, *P =* 0.011), and convexity (GLM: *χ* ^2^ = 12.7, *df* = 5, *P =* 0.026). Herbivory did not affect flower eccentricity (LM: *χ* ^2^ = 5.4, *df* = 5, *P =* 0.368). Petals had an average surface area of 0.13 cm^2^ and length and width of 0.5 × 0.3 cm. Herbivory affected the surface area of petals (Figure S3; GLM: *χ* ^2^ = 40.9, *df* = 5, *P* < 0.001), and petals of plants infested with *L. erysimi* or *D. radicum* were 7% larger than uninfested plants (Tukey's post hoc tests, *P =* 0.008 or *P =* 0.010, respectively). Herbivory affected several shape characteristics of petals (Figure S3), such as major chord length (GLM: *χ* ^2^ = 79.0, *df* = 5, *P* < 0.001), minor chord length (GLM: *χ* ^2^ = 37.3, *df* = 5, *P* < 0.001), aspect ratio (GLM: *χ* ^2^ = 52.3, *df* = 5, *P* < 0.001), and eccentricity (GLM: *χ* ^2^ = 59.0, *df* = 5, *P* < 0.001). Overall, petals of herbivore‐infested plants had smaller aspect ratios and eccentricity than had uninfested plants, caused by shorter (*A. rosae* and *B. brassicae*) or broader (*P. xylostella*, *L. erysimi*, *D. radicum*) petals. Despite changes in petal size caused by all herbivores, only flowers of plants infested with *P. xylostella* had larger display size and smaller solidity than had uninfested plants.

### 3.2 Effect of herbivore infestation on flower colour

The reflectance spectra of the top and base part of petals differed significantly (Figure S4; PERMANOVA: *R* ^2^ = 67.1, *df* = 1, *P* < 0.001). The reflectance spectra of the top part of petals, compared with the reflectance spectra of the base part of petals, included two maximum reflectance peaks at 326 and 351 nm. Because of these differences, we performed separate analyses for the effects of herbivory for the top and base part of petals. The colour of both top and base parts of petals of *B. nigra* was affected by herbivory (Figure S5; top—PERMANOVA: *R* ^2^ = 7.8, *df* = 5, *P* < 0.001; base—PERMANOVA: *R* = 9.7, *df* = 5, *P* < 0.001). The SVM models were accurate in identifying herbivore treatments of individual plants as defined by the training datasets, on the basis of the reflectance spectra of both the top and base parts of petals (Tables S2 and S3; top—SVM accuracy 89%, error rate 10%; base—SVM accuracy 83%, error rate 12%). This indicates herbivore‐species‐specific changes in the reflectance spectra of both the top and base parts of petals and thus significant differences between all treatments. In addition, the SVM models were accurate in identifying HFG treatments of individual plants as defined by the training datasets (Tables S2 and S3; top—SVM accuracy 98%, error rate 2%; base—SVM accuracy 92%, error rate 4%). This indicates HFG‐specific changes and thus significant differences between herbivores of different HFGs. This was confirmed by the ability of SVM models to assign plants infested with individual herbivore species to the correct HFGs and vice versa (Tables S2 and S3).

**Figure 1:**
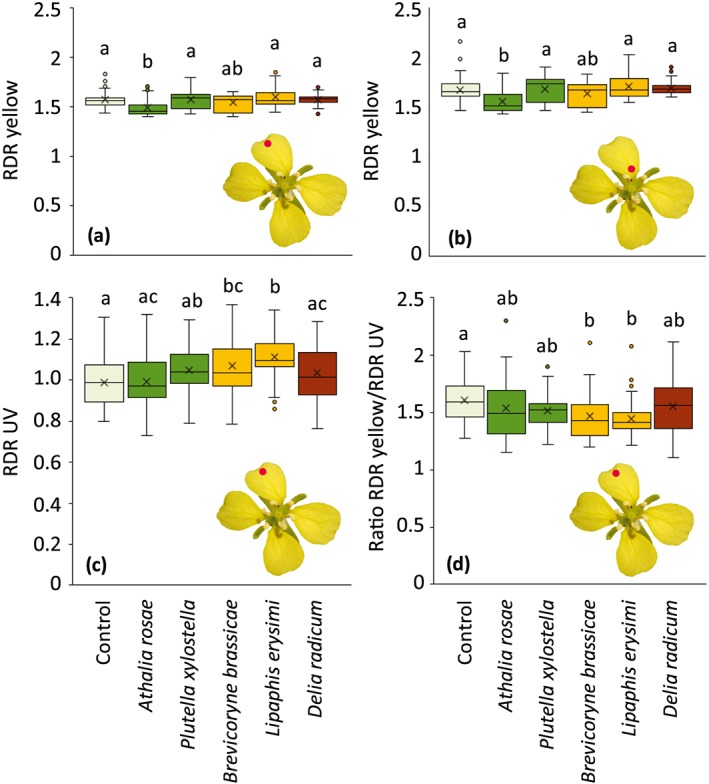
Relative diffuse reflection (RDR) of yellow (570–650 nm) and UV (310–370 nm) wavelengths by petals of uninfested *Brassica nigra* plants or plants infested with different herbivores. (a) Relative diffuse reflection of yellow of top parts of petals. (b) Relative diffuse reflection of yellow of base parts of petals. (c) Relative diffuse reflection of UV of top parts of petals. (d) Ratio RDR yellow/RDR UV of top parts of petals. Boxplots show median (line), mean (x), first and third quartiles, and minimum and maximum. Outliers (1.5 times the interquartile range below the first quartile or above the third quartile) are represented by circles. The red dot on the flower images indicates where measurements were taken (top or base), which was done after 7 days of herbivory. Number of replicates per herbivore treatment varied between six and eight plants. From each plant, six flowers were used, of which each petal was measured, both top and base parts. Letters above bars indicate significant differences at *α* = 0.05 based on Tukey's post hoc tests [Colour figure can be viewed at http://wileyonlinelibrary.com]

Spectral profiles of petals of *B. nigra* contained two regions of interest: the yellow/orange region (570–650 nm) and the UV region (310–370 nm). The relative diffuse reflectance of yellow/orange of the top part of petals was affected by herbivory (Figure 1; GLM: *χ* ^2^ = 28.48, *df* = 5, *P* < 0.001), as well as the base part of petals (Figure 1; GLM: *χ* ^2^ = 27.634, *df* = 5, *P* < 0.001). Petals of plants infested with *A. rosae* reflected 5–9% less yellow/orange than did all other treatments except plants infested with *B. brassicae* (Figure 1). The relative diffuse reflectance of UV of the top part of petals was affected by herbivory (Figure 1; GLM: *χ* ^2^ = 29.66, *df* = 5, *P* < 0.001). Flowers of plants infested with *B. brassicae* and *L. erysimi* reflected 8–13% more UV than did uninfested plants, and this resulted in a reduced yellow/UV ratio (Figure 1).

### 3.3 Effect of herbivore infestation on plant volatile emission

**Figure 2:**
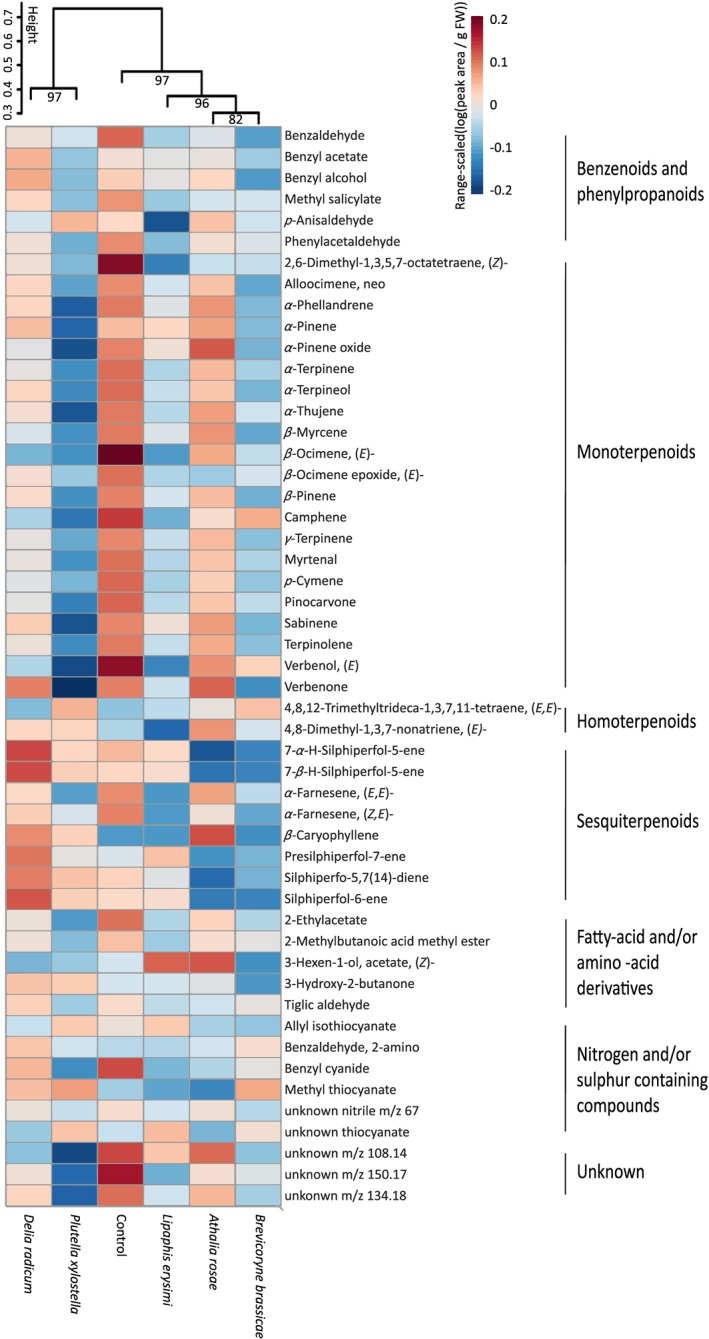
Dendrogram and heat map of the emission of volatile compounds of *Brassica nigra* plants infested with different herbivores or uninfested plants. Dendrogram clustering was performed using Ward's clustering algorithm with Euclidean distances. Values in the dendrogram are approximately unbiased probability values, where values ≥ 95 indicate significant differences. For the heat map, we used range‐scaled log‐transformed values of volatile emission (peak area/g FW) for each compound. Volatiles were collected after 7 days of herbivory. Number of replicates per herbivore treatment varied between seven and nine plants [Colour figure can be viewed at http://wileyonlinelibrary.com]

The volatile profile of flowering *B. nigra* plants consisted of 51 compounds of six major classes: benzenoids and phenylpropanoids, monoterpenoids, homoterpenoids, sesquiterpenoids, fatty‐acid and amino‐acid derivatives, and nitrogen‐containing compounds (see Table S4). Herbivore infestation, compared with uninfested plants, did not affect total volatile emission (Figure S6; LM: *χ* ^2^ = 4.84, *df* = 5, *P =* 0.436) and did not result in qualitative differences in the volatile profiles. Herbivore infestation resulted in quantitative differences in the volatile profiles of infested and uninfested plants (Figure 2). The largest difference was between plants infested with caterpillars of *P. xylostella* and the root herbivore *D. radicum*, and all other treatments. The volatile profile of uninfested plants differed from that of infested plants, where infested plants emitted lower amounts of benzenoids and phenylpropanoids (LM: *χ* ^2^ = 5.85, *df* = 1, *P =* 0.016) and tended to emit lower amounts of monoterpenoids (LM: *χ* ^2^ = 3.25, *df* = 1, *P =* 0.071). All herbivores differently affected the volatile profile by changing the emission of specific compounds, whereas the volatile profile of plants infested with *A. rosae* and *B. brassicae* and plants infested with *P. xylostella* and *D. radicum* were relatively similar (Figure 2). Moreover, herbivores differently affected volatile compound classes, which is evident from different clustering of the herbivores for each volatile compound class (Figure S7). Interestingly, herbivore‐induced changes in benzenoids and phenylpropanoids seem to match the observed herbivore‐induced changes in pollinator behaviour quite well (Table 1). We did not observe distinct clustering of volatile profiles on the basis of herbivore feeding guild or site. Thus, herbivore‐induced plant volatile profiles were specific for each attacking herbivore species within feeding guild or feeding site.

### 3.4 Effect of herbivore infestation on flower rewards

**Figure 3:**
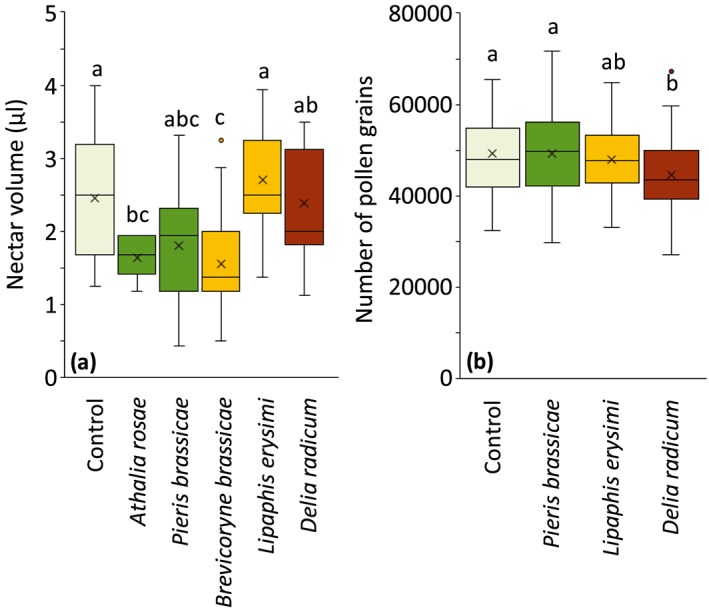
Nectar volume and number of pollen grains of uninfested *Brassica nigra* plants or plants infested with different herbivores. (a) Nectar volume of eight flowers of uninfested *B. nigra* plants or plants infested with different herbivores. Number of replicates per herbivore treatment varied between 14 and 15 plants. (b) Number of pollen grains of one flower of uninfested *B. nigra* plants or plants infested with different herbivores. Boxplots show median (line), mean (x), first and third quartiles, and minimum and maximum. Outliers (1.5 times the interquartile range below the first quartile or above the third quartile) are represented by circles. Nectar volume and number of pollen grains were measured after 7 days of herbivory. Number of replicates per herbivore treatment was 10 plants; per plant, we measured pollen for five flowers. Letters above bars indicate significant differences at *α* = 0.05 based on Tukey's post hoc tests [Colour figure can be viewed at http://wileyonlinelibrary.com]

Plants produced on average 0.26 μl of nectar and 47,768 pollen grains per flower. Nectar production of flowers was affected by herbivory (Figure 3; LM: *F* = 6.61, *df* = 5, *P* < 0.001). Plants infested with *A. rosae* or *B. brassicae* produced less nectar than did uninfested plants (Tukey's post hoc tests, *P =* 0.037 and *P =* 0.013, respectively), plants infested with *L. erysimi* (Tukey's post hoc tests, *P =* 0.002 and *P* < 0.001, respectively) or *D. radicum* (Tukey's post hoc tests, *P =* 0.072 and *P =* 0.029, respectively). The number of pollen grains produced by flowers was affected by herbivory (Figure 3; GLM: *χ* ^2^ = 10.09, *df* = 3, *P =* 0.018). Plants infested with *D. radicum* produced fewer pollen grains than did uninfested plants (Tukey's post hoc tests, *P =* 0.029) and plants infested with *P. brassicae* (Tukey's post hoc tests, *P =* 0.024). The average size of pollen grains did not differ between uninfested or herbivore‐infested plants (Figure S8; GLM: *χ* ^2^ = 0.18, *df* = 3, *P =* 0.981) nor did the pollen size distribution (Figure S9; *χ* ^2^ test, *P* = 1.000).

### 3.5 Effect of herbivore infestation on pollinator behaviour

**Figure 4:**
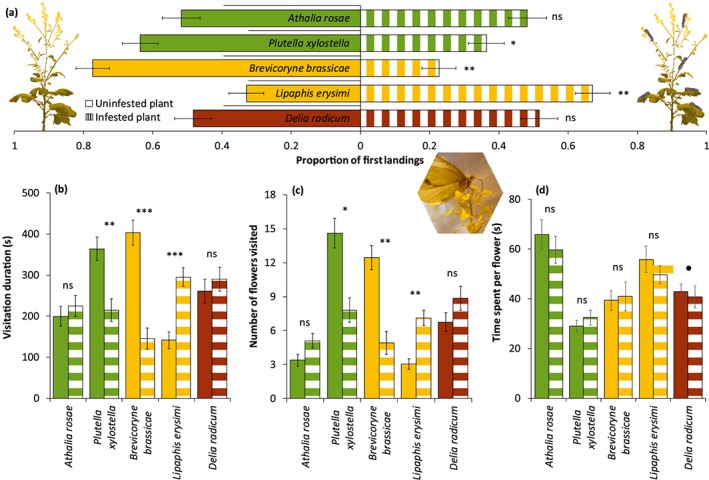
Preferences of the butterfly *Pieris brassicae* for uninfested *Brassica nigra* plants or plants infested with different herbivores. (a) Proportion of *P. brassicae* butterflies (mean ± *SE*) that first landed on flowers or leaves of *B. nigra* plants infested with different herbivores or uninfested plants. (b) Visitation duration (mean ± *SE*); (c) number of flowers visited (mean ± *SE*); and (d) time spent per flower (mean ± *SE*) by individual pollinators on infested or uninfested *B. nigra* plants. Butterfly behaviour was assessed after 7 days of herbivory. Number of replicates per herbivore treatment varied between 75 and 89 butterflies, and eight and 10 plant pairs. Asterisks above bars indicate significant differences with ***P < 0.001, **0.001 ≥ *P* < 0.01, *0.01 ≥ *P* ≤ 0.05, and ●0.05 > *P* < 0.1, based on Tukey's post hoc tests. Photograph shows a *P. brassicae* butterfly visiting flowers of *B. nigra*. Photograph credit: Quint Rusman [Colour figure can be viewed at http://wileyonlinelibrary.com]

We observed the behaviour of 902 responsive pollinators, with 108.4 hr of observation time, and over 8,000 flower visits; and these observations revealed that the behaviour of both butterflies and syrphid flies was affected by herbivore infestation. The direction of the effect depended on herbivore and pollinator species. Butterflies landed less frequently on plants infested with *P. xylostella* (GLMM: *z* = −2.25, *P =* 0.025) or *B. brassicae* (GLMM: *z* = −3.29, *P =* 0.001) and more frequently on plants infested with *L. erysimi* (GLMM: *z* = 3.13, *P =* 0.002) than on uninfested plants (Figure 4). Butterflies landed as frequently on plants infested with *A. rosae* (GLMM: *z* = −0.37, *P =* 0.715) or *D. radicum* (GLMM: *z* = 0.23, *P =* 0.816) as they did on uninfested plants. Herbivore infestation had the same effect on the duration of visitation and the number of flowers visited as on the landing preference for butterflies (Figure 4). Syrphid flies landed more frequently on plants infested with *L. erysimi* (GLMM: *z* = 2.09, *P =* 0.036) or *D. radicum* (GLMM: *z* = 2.23, *P =* 0.026) and tended to land less frequently on plants infested with *B. brassicae* (GLMM: *z* = −1.76, *P =* 0.079) than on uninfested plants (Figure S10). Syrphid flies landed as frequently on plants infested with *A. rosae* (GLMM: *z* = −0.62, *P =* 0.537) or *P. xylostella* (GLMM: *z* = −0.59, *P =* 0.557) as they did on uninfested plants. Herbivore infestation had the same effect on the duration of visitation and the number of flowers visited as on the landing preference for syrphid flies (Figure S10).

## 4 DISCUSSION

Our data show that responses of *B. nigra* to attack by different herbivores changed multiple flower traits and affected pollinator behaviour. Flower traits that most strongly changed in response to herbivory were flower morphology, flower colour, the composition of the volatile blend, and nectar production. For most traits, specificity is beyond feeding guild and site, and changes are herbivore‐species‐specific. The herbivore‐induced species‐specific changes in flower traits resulted in herbivore‐induced species‐specific changes in pollinator behaviour. The best predictors for the changes in pollinator behaviour were the combined changes in all flower traits, or changes in specific compounds within the volatile blend, especially benzenoids and phenylpropanoids. Changes in flower morphology and nectar production could explain pollinator behaviour in some cases, but not in others. Thus, flowers show extensive plasticity in response to antagonist herbivores, with contrasting effects on mutualist pollinators.

Flowering plants appear to be highly plastic in response to herbivory. Specificity in herbivore‐induced changes in flower traits yielded a unique phenotype to each herbivore attacker, with species‐specific effects on different pollinators. Our data do not support the predicted specificity of elicitation or effect on the basis of herbivore feeding guild and site (Rusman et al., 2018). This prediction was based on specificity of effect of pollinator functional groups to herbivory (Rusman et al., 2018), and specificity of elicitation of phytohormones involved in defence regulation (JA and SA) by herbivores from different feeding guilds and that use different feeding sites (Ali & Agrawal, 2012; Johnson et al., 2016; Thaler et al., 2012). Within pollinator functional groups, pollinator species can respond differently to plants exposed to the same herbivore (Hoffmeister et al., 2016). Apparently, specific effects on pollinator functional groups do not accurately predict specificity of elicitation of herbivore‐induced changes in flower traits. Moreover, specificity in elicitation of the phytohormones JA and SA might not accurately predict the induced phenotype, because of alternative signalling pathways and spatiotemporal modulators of the JA response (Erb et al., 2012). Despite effects of herbivore feeding guild on phytohormone induction and related gene transcription (Bidart‐Bouzat & Kliebenstein, 2011; De Vos et al., 2005), the resulting induced phenotype is at least partially herbivore‐species‐specific (Bidart‐Bouzat & Kliebenstein, 2011; Chung & Felton, 2011; Heidel & Baldwin, 2004; Travers‐Martin & Müller, 2007). The same seems to be true for the floral phenotype. For example, plant responses to root‐ and leaf‐chewing herbivores, which are both mediated by JA, had differential effects on flower traits such as petal size (Poveda, Steffan‐Dewenter, Scheu, & Tscharntke, 2005), flower abundance (Barber, Adler, & Bernardo, 2011), and flowering phenology (Poveda, Steffan‐Dewenter, Scheu, & Tscharntke, 2003). Application of JA reduced nectar production in *B. nigra*, whereas herbivory by *Pieris rapae* or *P. brassicae* caterpillars, which mainly induce JA, increased or had no effect on nectar production (Bruinsma et al., 2014; Bruinsma, IJdema, van Loon, & Dicke, 2008). Herbivory by two aphid species had differential effects on floral volatile emission (Pareja et al., 2012). Thus, specificity of herbivore‐induced floral traits is herbivore species‐specific and results in specificity of effects on pollinator visitation.

Herbivores induce changes in multiple components of the flower phenotype, which makes it difficult to disentangle the contribution of each component to the changes in pollinator behaviour. Plasticity in any trait might contribute to altered pollinator visitation. Moreover, because plants use multiple flower traits to attract pollinators (Junker & Parachnowitsch, 2015; Leonard, Dornhaus, & Papaj, 2012), the combination of all changes in flower traits might explain pollinator visitation best. Herbivore‐induced changes in individual flower traits may shift the relative importance of each flower trait within the complete floral phenotype for pollinator attraction (Lawson, Whitney, & Rands, 2017; Leonard et al., 2012). Still, some flower traits seem to be more plastic than others and may contribute more to pollinator attraction, more so when the trait provides information on floral rewards. Especially floral volatiles seem to undergo profound changes in response to herbivory (Cozzolino et al., 2015; Kessler & Halitschke, 2009; Lucas‐Barbosa et al., 2016; Pareja et al., 2012; Schiestl et al., 2014) and may explain changes in pollinator behaviour well (Kessler & Halitschke, 2009; Schiestl et al., 2014), although it is often unclear which individual compounds are used by pollinators (but see Knauer & Schiestl, 2015). Other flower traits like colour and morphology also change in response to herbivory and are important for pollinator attraction (Campbell, Bischoff, Lord, & Robertson, 2010; Hempel de Ibarra, Langridge, & Vorobyev, 2015; Strauss, Conner, & Rush, 1996). Flower volatiles, colour, and morphology can provide information on floral rewards for naïve pollinators (Gómez et al., 2008; Haverkamp, Bing, Badeke, Hansson, & Knaden, 2016; Raine & Chittka, 2007), conferring so‐called honest signals, which would predict adaptive responses of naïve pollinators to herbivore‐induced changes in flower volatiles, colour, or morphology. However, herbivory also resulted in changes in floral rewards, which could potentially disrupt honest signalling. In this study, the combined changes in all flower traits, or the induced changes in specific plant volatile compounds, especially in benzenoids and phenylpropanoids, seem to predict best the herbivore‐species‐specific changes in pollinator behaviour. Changes in flower morphology and nectar production could predict pollinator behaviour in some cases, but not in others. We did not investigate nectar and pollen composition, which may change in response to herbivory (Adler, Wink, Distl, & Lentz, 2006; Bruinsma et al., 2014) but do not always predict pollinator behaviour well (Carr, Haber, LeCroy, Lee, & Link, 2015; Vanderplanck et al., 2014). Although the mere presence of the herbivores on the plants could have affected pollinator behaviour directly, we never observed that pollinators would be especially attracted or repelled by herbivore‐infested inflorescences or the herbivores themselves in the greenhouse or field (Q. Rusman & D. Lucas‐Barbosa, personal observations). To identify the exact cause of herbivore‐induced changes on pollinator behaviour, future studies should manipulate individual flower traits and their combinations or use natural variation in flower traits to assess which traits most strongly affect flower visitation by different pollinator species. This will promote understanding of why flowers show such extensive plasticity to herbivore attack.

Here, our data show that responses of *B. nigra* plants to herbivores that regularly attack this plant species can have positive, negative, or neutral effects on pollinator behaviour and that different pollinators respond differently to herbivore‐induced changes in flower traits. This is supported by evidence from field studies, where multiple herbivores have been shown to differentially affect pollinator community composition, with positive, negative, or neutral effects on the visitation by pollinator species (Hoffmeister et al., 2016; Rusman et al., 2018). Changes in the behaviour of one or more pollinator groups and resulting changes in plant reproduction might confer considerable ecological costs of herbivore‐induced changes in flower traits. Interestingly, some plant species seem to be able to maintain overall pollinator visitation in the field (Barber et al., 2011; Hoffmeister et al., 2016 ; Lucas‐Barbosa et al., 2013 ; Rusman et al., 2018). Thus, despite the repellence of some pollinators by herbivore‐induced changes in flower traits, plants seem to compensate by attracting other pollinators. In this way, defences can be activated without compromising reproduction (Hoffmeister et al., 2016; Lucas‐Barbosa et al., 2013; Rusman et al., 2018), even when defence and flower traits are tightly linked (Jacobsen & Raguso, 2018; Lucas‐Barbosa, 2016). Alternatively, herbivore‐induced changes in flower traits as by‐products of the plant's inducible defensive responses may be maintained because they do not affect reproduction (Gould & Lewontin, 1979). Thus, ecological costs of flower plasticity in response to herbivory via disruptions of plant–pollinator interactions may be limited considering the entire pollinator community and in particular for generalized pollinator systems.

Our study shows that plant responses to herbivores feeding on the flowers, leaves, or roots have profound effects on the flower phenotype and pollinator visitation. Plant responses to any herbivore changed two or more traits, including flower morphological, chemical (colours and volatiles), and reward traits, and changes were herbivore species‐specific. This highlights the importance of exploring plant responses to multiple herbivores and pollinators and measuring multiple flower traits to reveal the underlying mechanisms of plant‐mediated herbivore–pollinator interactions. Herbivores potentially play a significant role as agents of selection on floral traits and plant reproduction via plant‐mediated interactions with pollinators.

## AUTHORS' CONTRIBUTION

Q.R., D.L.‐B., and E.H.P. planned and designed the study. Q.R. and F.N. collected the data. Q.R. analysed the data. G.P. provided the set‐up, assisted with measuring flower colour and morphology, and processed the flower images. Q.R., D.L.‐B., and E.H.P. interpreted the data and wrote the manuscript.

## Supporting information


**Table S1.** Specifications of the eight filters of the multispectral camera used to photograph flowers of herbivore‐infested and uninfested *Brassica nigra* plants.
**Fig. S1.** Number of flowers (a) and inflorescences (b) of *B. nigra* plants infested with different herbivores or uninfested plants.
**Fig. S2.** Morphometries for flowers of uninfested *B. nigra* plants and plants infested with different herbivores.
**Fig. S3.** Morphometries for petals of uninfested *B. nigra* plants and plants infested with different herbivores.
**Fig. S4.** Principal component analysis of the colour profile of the top (green) and base (red) part of petals of *B. nigra* plants.
**Fig. S5.** Reflectance spectra with relative diffuse reflectance of wavelengths (300‐700nm) of the top (a) and base (b) part of petals of *B. nigra* plants infested with different herbivores or uninfested plants.
**Table S2.** Confusion matrices of support vector machine classifiers for the reflectance spectra of top parts of petals of uninfested *B. nigra* plants of plants infested with different herbivores (a) or herbivore functional groups (HFGs) (b).
**Table S3.** Confusion matrices of support vector machine classifiers for the reflectance spectra of base parts of petals of uninfested *B. nigra* plants of plants infested with different herbivores (a) or herbivore functional groups (HFGs) (b).
**Fig. S6.** Total volatile emission of uninfested flowering *B. nigra* plants and plants infested with different herbivores.
**Table S4.** Volatile compounds of uninfested flowering *B. nigra* plants or plants infested with different herbivores.
**Fig. S7.** Dendrogram and heat map of the emission of volatile compounds for each compound class of *Brassica nigra* plants infested with different herbivores or uninfested plants.
**Fig. S8.** Size of pollen grains of uninfested *B. nigra* plants or plants infested with different herbivores.
**Fig. S9.** Size distribution of pollen grains of uninfested *B. nigra* plants or plants infested with different herbivores.
**Fig. S10.** Preference of the syrphid fly *Episyrphus balteatus* for uninfested *B. nigra* plants or plants infested with different herbivores.

## References

[pce13520-bib-0080] Adams, R. P. (1995). Identification of essential oil components by gas chromatography/mass spectrometry (4th ed.). Carol Stream: Allurum Publishing Corporation.

[pce13520-bib-0001] Adler, L. S. , Wink, M. , Distl, M. , & Lentz, A. J. (2006). Leaf herbivory and nutrients increase nectar alkaloids. Ecology Letters, 9, 960–967. 10.1111/j.1461-0248.2006.00944.x 16913940

[pce13520-bib-0002] Agrawal, A. A. (2011). Current trends in the evolutionary ecology of plant defence. Functional Ecology, 25, 420–432. 10.1111/j.1365-2435.2010.01796.x

[pce13520-bib-0003] Akter, A. , Biella, P. , & Klecka, J. (2017). Effects of small‐scale clustering of flowers on pollinator foraging behaviour and flower visitation rate. PLoS ONE, 12, e0187976 10.1371/journal.pone.0187976 29136042 PMC5685580

[pce13520-bib-0004] Ali, J. G. , & Agrawal, A. A. (2012). Specialist versus generalist insect herbivores and plant defense. Trends in Plant Science, 17, 293–302. 10.1016/j.tplants.2012.02.006 22425020

[pce13520-bib-0005] Avanci, N. , Luche, D. , Goldman, G. , & Goldman, M. (2010). Jasmonates are phytohormones with multiple functions, including plant defense and reproduction. Genetics and Molecular Research, 9, 484–505. 10.4238/vol9-1gmr754 20391333

[pce13520-bib-0006] Barber, N. A. , Adler, L. S. , & Bernardo, H. L. (2011). Effects of above‐and belowground herbivory on growth, pollination, and reproduction in cucumber. Oecologia, 165, 377–386. 10.1007/s00442-010-1779-x 20859750

[pce13520-bib-0007] Bates, D. , Maechler, M. , Bolker, B. , & Walker, S. (2015). Fitting linear mixed‐effects models using lme4. Journal of Statistical Software, 67, 1–48.

[pce13520-bib-0008] Bidart‐Bouzat, M. G. , & Kliebenstein, D. (2011). An ecological genomic approach challenging the paradigm of differential plant responses to specialist versus generalist insect herbivores. Oecologia, 167, 677–689. 10.1007/s00442-011-2015-z 21625984

[pce13520-bib-0009] Botto‐Mahan, C. , Ramírez, P. A. , Ossa, C. G. , Medel, R. , Ojeda‐Camacho, M. , & González, A. V. (2011). Floral herbivory affects female reproductive success and pollinator visitation in the perennial herb *Alstroemeria ligtu* (Alstroemeriaceae). International Journal of Plant Sciences, 172, 1130–1136. 10.1086/662029

[pce13520-bib-0010] Brioudes, F. , Joly, C. , Szécsi, J. , Varaud, E. , Leroux, J. , Bellvert, F. , … Bendahmane, M. (2009). Jasmonate controls late development stages of petal growth in *Arabidopsis thaliana* . The Plant Journal, 60, 1070–1080. 10.1111/j.1365-313X.2009.04023.x 19765234

[pce13520-bib-0011] Bruinsma, M. , IJdema, H. , van Loon, J. J. A. , & Dicke, M. (2008). Differential effects of jasmonic acid treatment of *Brassica nigra* on the attraction of pollinators, parasitoids, and butterflies. Entomologia Experimentalis et Applicata, 128, 109–116. 10.1111/j.1570-7458.2008.00695.x

[pce13520-bib-0012] Bruinsma, M. , Lucas‐Barbosa, D. , ten Broeke, C. J. , van Dam, N. M. , van Beek, T. A. , Dicke, M. , & van Loon, J. J. A. (2014). Folivory affects composition of nectar, floral odor and modifies pollinator behavior. Journal of Chemical Ecology, 40, 39–49. 10.1007/s10886-013-0369-x 24317664

[pce13520-bib-0013] Campbell, D. R. , Bischoff, M. , Lord, J. M. , & Robertson, A. W. (2010). Flower color influences insect visitation in alpine New Zealand. Ecology, 91, 2638–2649. 10.1890/09-0941.1 20957958

[pce13520-bib-0014] Carr, D. E. , Haber, A. I. , LeCroy, K. A. , Lee, D. A. E. , & Link, R. I. (2015). Variation in reward quality and pollinator attraction: The consumer does not always get it right. AoB PLANTS, 7, plv034. 25858692 10.1093/aobpla/plv034 PMC4417137

[pce13520-bib-0015] Chautá, A. , Whitehead, S. , Amaya‐Márquez, M. , & Poveda, K. (2017). Leaf herbivory imposes fitness costs mediated by hummingbird and insect pollinators. PLoS ONE, 12, e0188408 10.1371/journal.pone.0188408 29211805 PMC5718403

[pce13520-bib-0016] Chrétien, L. T. S. , David, A. , Daikou, E. , Boland, W. , Gershenzon, J. , Giron, D. , … Lucas‐Barbosa, D. (2018). Caterpillars induce jasmonates in flowers and alter plant responses to a second attacker. New Phytologist, 217, 1279–1291. 10.1111/nph.14904 29207438 PMC5814890

[pce13520-bib-0017] Chung, S. H. , & Felton, G. W. (2011). Specificity of induced resistance in tomato against specialist lepidopteran and coleopteran species. Journal of Chemical Ecology, 37, 378–386. 10.1007/s10886-011-9937-0 21455676

[pce13520-bib-0018] Cortes, C. , & Vapnik, V. (1995). Support‐vector networks. Machine Learning, 20, 273–297. 10.1007/BF00994018

[pce13520-bib-0019] Courtney, S. P. , Hill, C. J. , & Westerman, A. (1982). Pollen carried for long periods by butterflies. Oikos, 38, 260–263. 10.2307/3544030

[pce13520-bib-0020] Cozzolino, S. , Fineschi, S. , Litto, M. , Scopece, G. , Trunschke, J. , & Schiestl, F. P. (2015). Herbivory increases fruit set in *Silene latifolia*: A consequence of induced pollinator‐attracting floral volatiles? Journal of Chemical Ecology, 41, 622–630. 10.1007/s10886-015-0597-3 26085479

[pce13520-bib-0021] De Vos, M. , van Oosten, V. R. , van Poecke, R. M. , van Pelt, J. A. , Pozo, M. J. , Mueller, M. J. , … Pieterse, C. M. J. (2005). Signal signature and transcriptome changes of *Arabidopsis* during pathogen and insect attack. Molecular Plant‐Microbe Interactions, 18, 923–937. 10.1094/MPMI-18-0923 16167763

[pce13520-bib-0022] Dicke, M. , & van Loon, J. J. A. (2014). Chemical ecology of phytohormones: How plants integrate responses to complex and dynamic environments. Journal of Chemical Ecology, 40, 653–656. 10.1007/s10886-014-0479-0 25037238

[pce13520-bib-0023] Erb, M. , Meldau, S. , & Howe, G. A. (2012). Role of phytohormones in insect‐specific plant reactions. Trends in Plant Science, 17, 250–259. 10.1016/j.tplants.2012.01.003 22305233 PMC3346861

[pce13520-bib-0024] Fife, D. (2014) fifer: A collection of miscellaneous functions.

[pce13520-bib-0025] Gómez, J. M. , Bosch, J. , Perfectti, F. , Fernández, J. D. , Abdelaziz, M. , & Camacho, J. P. M. (2008). Association between floral traits and rewards in *Erysimum mediohispanicum* (Brassicaceae). Annals of Botany, 101, 1413–1420. 10.1093/aob/mcn053 18424472 PMC2710261

[pce13520-bib-0026] Gould, S. J. , & Lewontin, R. C. (1979). The spandrels of San Marco and the Panglossian paradigm: A critique of the adaptationist programme. Proceedings of the Royal Society of London, Series B: Biological Sciences, 205, 581–598. 10.1098/rspb.1979.0086 42062

[pce13520-bib-0027] Gronquist, M. , Bezzerides, A. , Attygalle, A. , Meinwald, J. , Eisner, M. , & Eisner, T. (2001). Attractive and defensive functions of the ultraviolet pigments of a flower (*Hypericum calycinum*). Proceedings of the National Academy of Sciences, 98, 13745–13750. 10.1073/pnas.231471698 PMC61112 11707571

[pce13520-bib-0028] Haverkamp, A. , Bing, J. , Badeke, E. , Hansson, B. S. , & Knaden, M. (2016). Innate olfactory preferences for flowers matching proboscis length ensure optimal energy gain in a hawkmoth. Nature Communications, 7, 11644 10.1038/ncomms11644 PMC4869250 27173441

[pce13520-bib-0029] Heidel, A. , & Baldwin, I. (2004). Microarray analysis of salicylic acid‐and jasmonic acid‐signalling in responses of *Nicotiana attenuata* to attack by insects from multiple feeding guilds. Plant, Cell & Environment, 27, 1362–1373. 10.1111/j.1365-3040.2004.01228.x

[pce13520-bib-0030] Heil, M. (2002). Ecological costs of induced resistance. Current Opinion in Plant Biology, 5, 345–350. 10.1016/S1369-5266(02)00267-4 12179969

[pce13520-bib-0031] Hempel de Ibarra, N. , Langridge, K. V. , & Vorobyev, M. (2015). More than colour attraction: Behavioural functions of flower patterns. Current Opinion in Insect Science, 12, 64–70. 10.1016/j.cois.2015.09.005 27064650 PMC4804388

[pce13520-bib-0032] Hoffmeister, M. , Wittköpper, N. , & Junker, R. R. (2016). Herbivore‐induced changes in flower scent and morphology affect the structure of flower‐visitor networks but not plant reproduction. Oikos, 125, 1241–1249. 10.1111/oik.02988

[pce13520-bib-0033] Hothorn, T. , Bretz, F. , & Westfall, P. (2008). Simultaneous inference in general parametric models. Biometrical Journal, 50, 346–363. 10.1002/bimj.200810425 18481363

[pce13520-bib-0034] Jacobsen, D. J. , & Raguso, R. A. (2018). Lingering effects of herbivory and plant defenses on pollinators. Current Biology, 28, R1164–R1169. 10.1016/j.cub.2018.08.010 30300606

[pce13520-bib-0035] Jauker, F. , & Wolters, V. (2008). Hover flies are efficient pollinators of oilseed rape. Oecologia, 156, 819–823. 10.1007/s00442-008-1034-x 18438687

[pce13520-bib-0036] Johnson, S. N. , Erb, M. , & Hartley, S. E. (2016). Roots under attack: Contrasting plant responses to below‐ and aboveground insect herbivory. New Phytologist, 210, 413–418. 10.1111/nph.13807 26781566

[pce13520-bib-0037] Junker, R. R. , & Parachnowitsch, A. L. (2015). Working towards a holistic view on flower traits—How floral scents mediate plant–animal interactions in concert with other floral characters. Journal of the Indian Institute of Science, 95, 43–68.

[pce13520-bib-0038] Karban, R. (2011). The ecology and evolution of induced resistance against herbivores. Functional Ecology, 25, 339–347. 10.1111/j.1365-2435.2010.01789.x

[pce13520-bib-0039] Karban, R. , & Baldwin, I. T. (1997). Induced responses to herbivory. Chicago: University of Chicago Press.

[pce13520-bib-0040] Kessler, A. (2015). The information landscape of plant constitutive and induced secondary metabolite production. Current Opinion in Insect Science, 8, 47–53. 10.1016/j.cois.2015.02.002 32846677

[pce13520-bib-0041] Kessler, A. , & Halitschke, R. (2009). Testing the potential for conflicting selection on floral chemical traits by pollinators and herbivores: predictions and case study. Functional Ecology, 23, 901–912. 10.1111/j.1365-2435.2009.01639.x

[pce13520-bib-0042] Knauer, A. , & Schiestl, F. (2015). Bees use honest floral signals as indicators of reward when visiting flowers. Ecology Letters, 18, 135–143. 10.1111/ele.12386 25491788

[pce13520-bib-0043] Lawson, D. A. , Whitney, H. M. , & Rands, S. A. (2017). Colour as a backup for scent in the presence of olfactory noise: testing the efficacy backup hypothesis using bumblebees (*Bombus terrestris*). Royal Society Open Science, 4, 170996 10.1098/rsos.170996 29291092 PMC5717666

[pce13520-bib-0044] Leonard, A. S. , Dornhaus, A. , & Papaj, D. R. (2012). Why are floral signals complex? An outline of functional hypotheses In Patiny S. (Ed.), Evolution of plant–pollinator relationships (pp. 261–282). Cambridge: Cambridge University Press.

[pce13520-bib-0045] Liao, K. , Gituru, R. W. , Guo, Y.‐H. , & Wang, Q.‐F. (2013). Effects of floral herbivory on foraging behaviour of bumblebees and female reproductive success in *Pedicularis gruina* (Orobanchaceae). Flora ‐ Morphology, Distribution, Functional Ecology of Plants, 208, 562–569. 10.1016/j.flora.2013.08.007

[pce13520-bib-0046] Lucas‐Barbosa, D. (2016). Integrating studies on plant–pollinator and plant–herbivore interactions. Trends in Plant Science, 21, 125–133. 10.1016/j.tplants.2015.10.013 26598297

[pce13520-bib-0047] Lucas‐Barbosa, D. , Poelman, E. H. , Aartsma, Y. , Snoeren, T. A. L. , van Loon, J. J. A. , & Dicke, M. (2014). Caught between parasitoids and predators—Survival of a specialist herbivore on leaves and flowers of mustard plants. Journal of Chemical Ecology, 40, 621–631. 10.1007/s10886-014-0454-9 24888744

[pce13520-bib-0048] Lucas‐Barbosa, D. , Sun, P. , Hakman, A. , van Beek, T. A. , van Loon, J. J. A. , & Dicke, M. (2016). Visual and odour cues: Plant responses to pollination and herbivory affect the behaviour of flower visitors. Functional Ecology, 30, 431–441. 10.1111/1365-2435.12509

[pce13520-bib-0049] Lucas‐Barbosa, D. , van Loon, J. J. A. , & Dicke, M. (2011). The effects of herbivore‐induced plant volatiles on interactions between plants and flower‐visiting insects. Phytochemistry, 72, 1647–1654. 10.1016/j.phytochem.2011.03.013 21497866

[pce13520-bib-0050] Lucas‐Barbosa, D. , van Loon, J. J. A. , Gols, R. , Beek, T. A. , & Dicke, M. (2013). Reproductive escape: annual plant responds to butterfly eggs by accelerating seed production. Functional Ecology, 27, 245–254. 10.1111/1365-2435.12004

[pce13520-bib-0051] Martínez, C. , Pons, E. , Prats, G. , & León, J. (2004). Salicylic acid regulates flowering time and links defence responses and reproductive development. The Plant Journal, 37, 209–217. 10.1046/j.1365-313X.2003.01954.x 14690505

[pce13520-bib-0052] Meyer, D. , Dimitriadou, E. , Hornik, K. , Weingessel, A. , & Leisch, F. (2017). e1071: Misc functions of the Department of Statistics, Probability Theory Group (Formerly: E1071), TU Wien. *Comprehensive R Archive Network (CRAN)*.

[pce13520-bib-0053] Muhlemann, J. K. , Klempien, A. , & Dudareva, N. (2014). Floral volatiles: From biosynthesis to function. Plant, Cell & Environment, 37, 1936–1949. 10.1111/pce.12314 24588567

[pce13520-bib-0054] Oksanen, J. , Kindt, R. , Legendre, P. , O'Hara, B. , Stevens, M. H. H. , Oksanen, M. J. , & Suggests, M. (2007). The vegan package. Community Ecology Package, 10, 631–637.

[pce13520-bib-0055] Ollerton, J. , Winfree, R. , & Tarrant, S. (2011). How many flowering plants are pollinated by animals? Oikos, 120, 321–326. 10.1111/j.1600-0706.2010.18644.x

[pce13520-bib-0056] Pareja, M. , Qvarfordt, E. , Webster, B. , Mayon, P. , Pickett, J. , Birkett, M. , & Glinwood, R. (2012). Herbivory by a phloem‐feeding insect inhibits floral volatile production. PLoS ONE, 7, e31971 10.1371/journal.pone.0031971 22384116 PMC3285634

[pce13520-bib-0057] Poelman, E. H. (2015). From induced resistance to defence in plant–insect interactions. Entomologia Experimentalis et Applicata, 157, 11–17. 10.1111/eea.12334

[pce13520-bib-0058] Poelman, E. H. , & Kessler, A. (2016). Keystone herbivores and the evolution of plant defenses. Trends in Plant Science, 21, 477–485. 10.1016/j.tplants.2016.01.007 26832946

[pce13520-bib-0059] Poveda, K. , Steffan‐Dewenter, I. , Scheu, S. , & Tscharntke, T. (2003). Effects of below‐ and above‐ground herbivores on plant growth, flower visitation and seed set. Oecologia, 135, 601–605. 10.1007/s00442-003-1228-1 16228257

[pce13520-bib-0060] Poveda, K. , Steffan‐Dewenter, I. , Scheu, S. , & Tscharntke, T. (2005). Effects of decomposers and herbivores on plant performance and aboveground plant–insect interactions. Oikos, 108, 503–510. 10.1111/j.0030-1299.2005.13664.x

[pce13520-bib-0061] Radhika, V. , Kost, C. , Boland, W. , & Heil, M. (2010). The role of jasmonates in floral nectar secretion. PLoS ONE, 5, e9265 10.1371/journal.pone.0009265 20174464 PMC2824824

[pce13520-bib-0062] Raine, N. E. , & Chittka, L. (2007). The adaptive significance of sensory bias in a foraging context: Floral colour preferences in the bumblebee *Bombus terrestris* . PLoS ONE, 2, e556 10.1371/journal.pone.0000556 17579727 PMC1891088

[pce13520-bib-0063] Raskin, I. , Ehmann, A. , Melander, W. R. , & Meeuse, B. J. D. (1987). Salicylic acid: A natural inducer of heat production in *Arum* lilies. Science, 237, 1601–1602. 10.1126/science.237.4822.1601 17834449

[pce13520-bib-0064] Rivas‐San Vicente, M. , & Plasencia, J. (2011). Salicylic acid beyond defence: Its role in plant growth and development. Journal of Experimental Botany, 62, 3321–3338. 10.1093/jxb/err031 21357767

[pce13520-bib-0065] Rusman, Q. , Lucas‐Barbosa, D. , & Poelman, E. H. (2018). Dealing with mutualists and antagonists: Specificity of plant‐mediated interactions between herbivores and flower visitors, and consequences for plant fitness. Functional Ecology, 32, 1022–1035. 10.1111/1365-2435.13035

[pce13520-bib-0066] Schiestl, F. P. , Kirk, H. , Bigler, L. , Cozzolino, S. , & Desurmont, G. A. (2014). Herbivory and floral signaling: Phenotypic plasticity and tradeoffs between reproduction and indirect defense. New Phytologist, 203, 257–266. 10.1111/nph.12783 24684288

[pce13520-bib-0067] Strauss, S. Y. (1997). Floral characters link herbivores, pollinators, and plant fitness. Ecology, 78, 1640–1645. 10.1890/0012-9658(1997)078[1640:FCLHPA]2.0.CO;2

[pce13520-bib-0068] Strauss, S. Y. , Conner, J. K. , & Rush, S. L. (1996). Foliar herbivory affects floral characters and plant attractiveness to pollinators: Implications for male and female plant fitness. American Naturalist, 147, 1098–1107. 10.1086/285896

[pce13520-bib-0069] Strauss, S. Y. , Rudgers, J. A. , Lau, J. A. , & Irwin, R. E. (2002). Direct and ecological costs of resistance to herbivory. Trends in Ecology & Evolution, 17, 278–285. 10.1016/S0169-5347(02)02483-7

[pce13520-bib-0070] Suzuki, R. , & Shimodaira, H. (2006). Pvclust: an R package for assessing the uncertainty in hierarchical clustering. Bioinformatics, 22, 1540–1542. 10.1093/bioinformatics/btl117 16595560

[pce13520-bib-0071] Thaler, J. S. , Humphrey, P. T. , & Whiteman, N. K. (2012). Evolution of jasmonate and salicylate signal crosstalk. Trends in Plant Science, 17, 260–270. 10.1016/j.tplants.2012.02.010 22498450

[pce13520-bib-0072] Travers‐Martin, N. , & Müller, C. (2007). Specificity of induction responses in *Sinapis alba* L. and their effects on a specialist herbivore. Journal of Chemical Ecology, 33, 1582–1597. 10.1007/s10886-007-9322-1 17587140

[pce13520-bib-0073] Vanderplanck, M. , Moerman, R. , Rasmont, P. , Lognay, G. , Wathelet, B. , Wattiez, R. , & Michez, D. (2014). How does pollen chemistry impact development and feeding behaviour of polylectic bees? PLoS ONE, 9, e86209 10.1371/journal.pone.0086209 24465963 PMC3897652

[pce13520-bib-0074] Wilcock, C. , & Neiland, R. (2002). Pollination failure in plants: Why it happens and when it matters. Trends in Plant Science, 7, 270–277. 10.1016/S1360-1385(02)02258-6 12049924

[pce13520-bib-0075] Xia, J. , Sinelnikov, I. V. , Han, B. , & Wishart, D. S. (2015). MetaboAnalyst 3.0—Making metabolomics more meaningful. Nucleic Acids Research, 43, 251–257. 10.1093/nar/gkv380 PMC4489235 25897128

[pce13520-bib-0076] Yuan, Z. , & Zhang, D. (2015). Roles of jasmonate signalling in plant inflorescence and flower development. Current Opinion in Plant Biology, 27, 44–51. 10.1016/j.pbi.2015.05.024 26125498

[pce13520-bib-0077] Zeileis, A. , & Hothorn, T. (2002). Diagnostic checking in regression relationships. R News, 2, 7–10.

