# Supplementary material for: Floral plasticity: Herbivore‐species‐specific‐induced changes in flower traits with contrasting effects on pollinator visitation
Source: Plant Cell Environ. 2019 Mar 8;42(6):1882–96. doi: 10.1111/pce.13520 (PMC6850075; doi:10.1111/pce.13520)
Supplement: Supplementary file 1 — Table S1. Specifications of the eight filters of the multispectral camera used to photograph flowers of herbivore‐infested and uninfested Brassica nigra plants. Fig. S1. Number of flowers (a) and inflorescences (b) of B. nigra plants infested with different herbivores or uninfested plants. Fig. S2. Morphometries for flowers of uninfested B. nigra plants and plants infested with different herbivores. Fig. S3. Morphometries for petals of uninfested B. nigra plants and plants infested with different herbivores. Fig. S4. Principal component analysis of the colour profile of the top (green) and base (red) part of petals of B. nigra plants. Fig. S5. Reflectance spectra with relative diffuse reflectance of wavelengths (300‐700nm) of the top (a) and base (b) part of petals of B. nigra plants infested with different herbivores or uninfested plants. Table S2. Confusion matrices of support vector machine classifiers for the reflectance spectra of top parts of petals of uninfested B. nigra plants of plants infested with different herbivores (a) or herbivore functional groups (HFGs) (b). Table S3. Confusion matrices of support vector machine classifiers for the reflectance spectra of base parts of petals of uninfested B. nigra plants of plants infested with different herbivores (a) or herbivore functional groups (HFGs) (b). Fig. S6. Total volatile emission of uninfested flowering B. nigra plants and plants infested with different herbivores. Table S4. Volatile compounds of uninfested flowering B. nigra plants or plants infested with different herbivores. Fig. S7. Dendrogram and heat map of the emission of volatile compounds for each compound class of Brassica nigra plants infested with different herbivores or uninfested plants. Fig. S8. Size of pollen grains of uninfested B. nigra plants or plants infested with different herbivores. Fig. S9. Size distribution of pollen grains of uninfested B. nigra plants or plants infested with different herbivores. Fig. S10. Preference of the [file PCE-42-1882-s001.pdf]

Supporting Information

Floral plasticity: Herbivore-species-specific induced changes in flower traits with contrasting effects on pollinator visitation.

Quint Rusman[1], Erik H. Poelman[1], Farzana Nowrin[1,2], Gerrit Polder[3], and Dani Lucas-Barbosa[1,4]

[1]Laboratory of Entomology, Wageningen University, P.O. Box 16, 6700 AA Wageningen, The Netherlands

[2]Current address: Entomology Division, Bangladesh Rice Research Institute, Gazipur-1701, Bangladesh

[3] Wageningen UR Greenhouse Horticulture, P.O. Box 644, 6700 AP Wageningen, The Netherlands

[4] Current address: Bio-communication & Ecology, ETH Zürich, Schmelzbergstrasse 9, 8092 Zürich, Switzerland

Corresponding author: quint.rusman@wur.nl

The following figures and tables are included in the supporting information:

Table S1. Specifications of the eight filters of the multispectral camera used to photograph flowers of herbivore-infested and uninfested *Brassica nigra* plants.

Fig. S1. Number of flowers (a) and inflorescences (b) of *B. nigra* plants infested with different herbivores or uninfested plants.

Fig. S2. Morphometries for flowers of uninfested *B. nigra* plants and plants infested with different herbivores.

Fig. S3. Morphometries for petals of uninfested *B. nigra* plants and plants infested with different herbivores.

Fig. S4. Principal component analysis of the colour profile of the top (green) and base (red) part of petals of *B. nigra* plants.

Fig. S5. Reflectance spectra with relative diffuse reflectance of wavelengths (300-700nm) of the top (a) and base (b) part of petals of *B. nigra* plants infested with different herbivores or uninfested plants.

Table S2. Confusion matrices of support vector machine classifiers for the reflectance spectra of top parts of petals of uninfested *B. nigra* plants of plants infested with different herbivores (a) or herbivore functional groups (HFGs) (b).

Table S3. Confusion matrices of support vector machine classifiers for the reflectance spectra of base parts of petals of uninfested *B. nigra* plants of plants infested with different herbivores (a) or herbivore functional groups (HFGs) (b).

Fig. S6. Total volatile emission of uninfested flowering *B. nigra* plants and plants infested with different herbivores.

Table S4. Volatile compounds of uninfested flowering *B. nigra* plants or plants infested with different herbivores.

Fig. S7. Dendrogram and heat map of the emission of volatile compounds for each compound class of *Brassica nigra* plants infested with different herbivores or uninfested plants.

Fig. S8. Size of pollen grains of uninfested *B. nigra* plants or plants infested with different herbivores.

Fig. S9. Size distribution of pollen grains of uninfested *B. nigra* plants or plants infested with different herbivores.

Fig. S10. Preference of the syrphid fly *Episyrphus balteatus* for uninfested *B. nigra* plants or plants infested with different herbivores.

Table S1. Specifications of the eight filters of the multispectral camera used to photograph flowers of herbivore-infested and uninfested *Brassica nigra* plants.

Filter	Wavelength [nm]	Bandwidth [nm]	Exposure [ms]	Gain [dB]
1	425	50	20	100
2	466	21	30	0
3	500	20	10	0
4	542	10	7	0
5	570	50	1	0
6	601	13	3	0
7	640	20	3	0
8	680	20	3	0

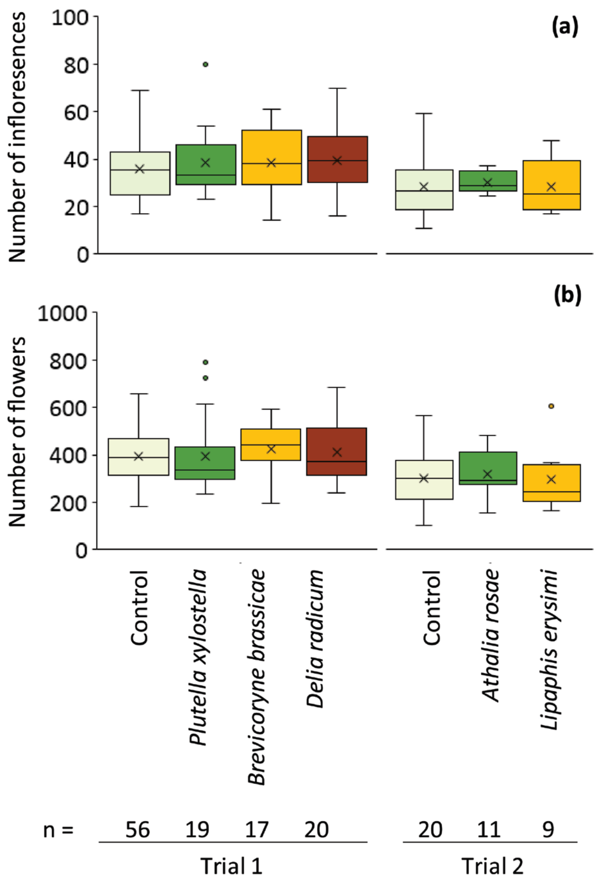

Fig. S1 Number of flowers (a) and inflorescences (b) of *Brassica nigra* plants infested with different herbivores or uninfested plants. Boxplots show median (line), mean (x), 1st and 3rd quartiles, minimum and maximum. Outliers (1.5 times the interquartile range below the 1st or above the 3rd quartile) are represented by circles. Flowers and inflorescences were counted after seven days of herbivory.

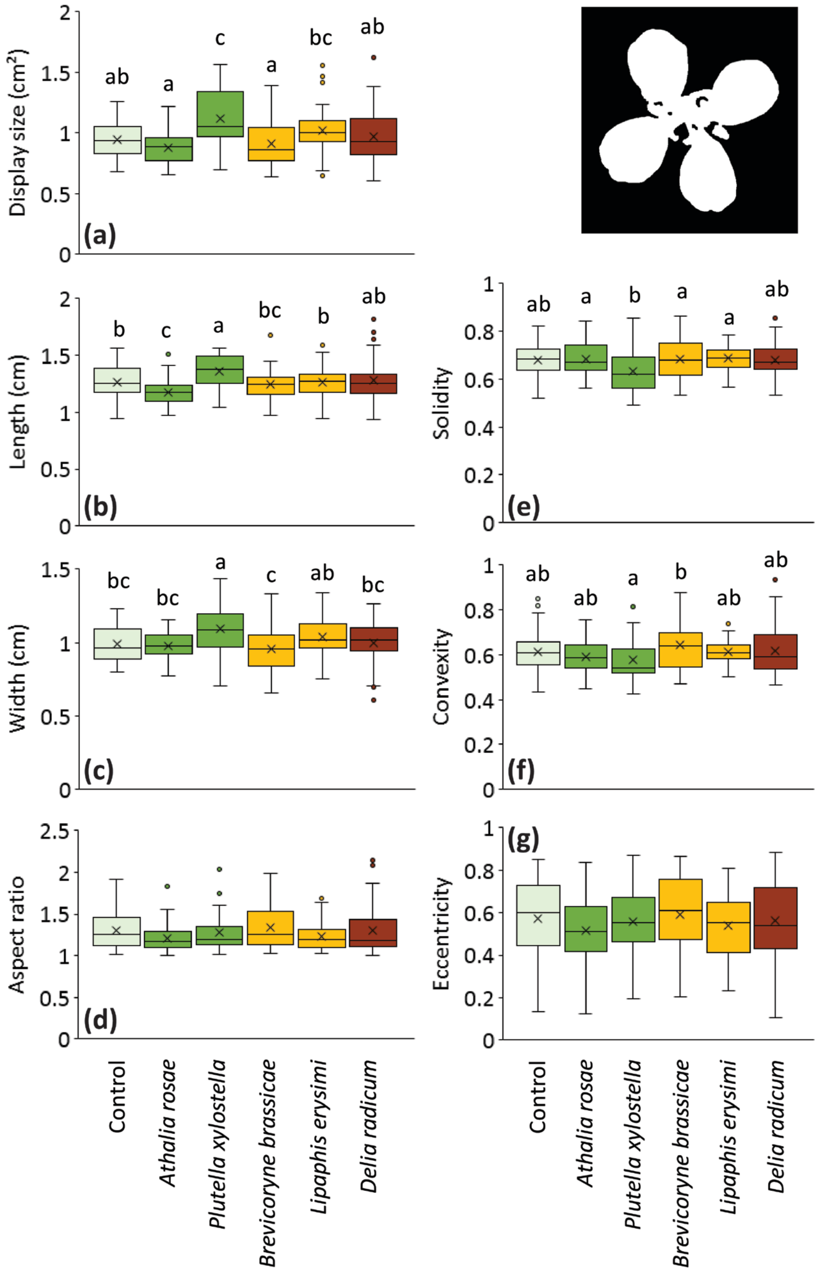

Fig. S2 Morphometry for flowers of uninfested *Brassica nigra* plants and plants infested with different herbivores. We measured a) display size, b) length, c) width, d) aspect ratio, e) solidity, f) convexity, and g) eccentricity. Boxplots show median (line), mean (x), 1st and 3rd quartiles, minimum and maximum. Outliers (1.5 times the interquartile range below the 1st or above the 3rd quartile) are represented by circles. Measurements were taken after seven days of herbivory. Number of replicates per herbivore treatment varied between seven and eight plants, and six flowers were measured from each plant. Letters above bars indicate significant differences at α = 0.05 based on Tukey's *post hoc* tests.

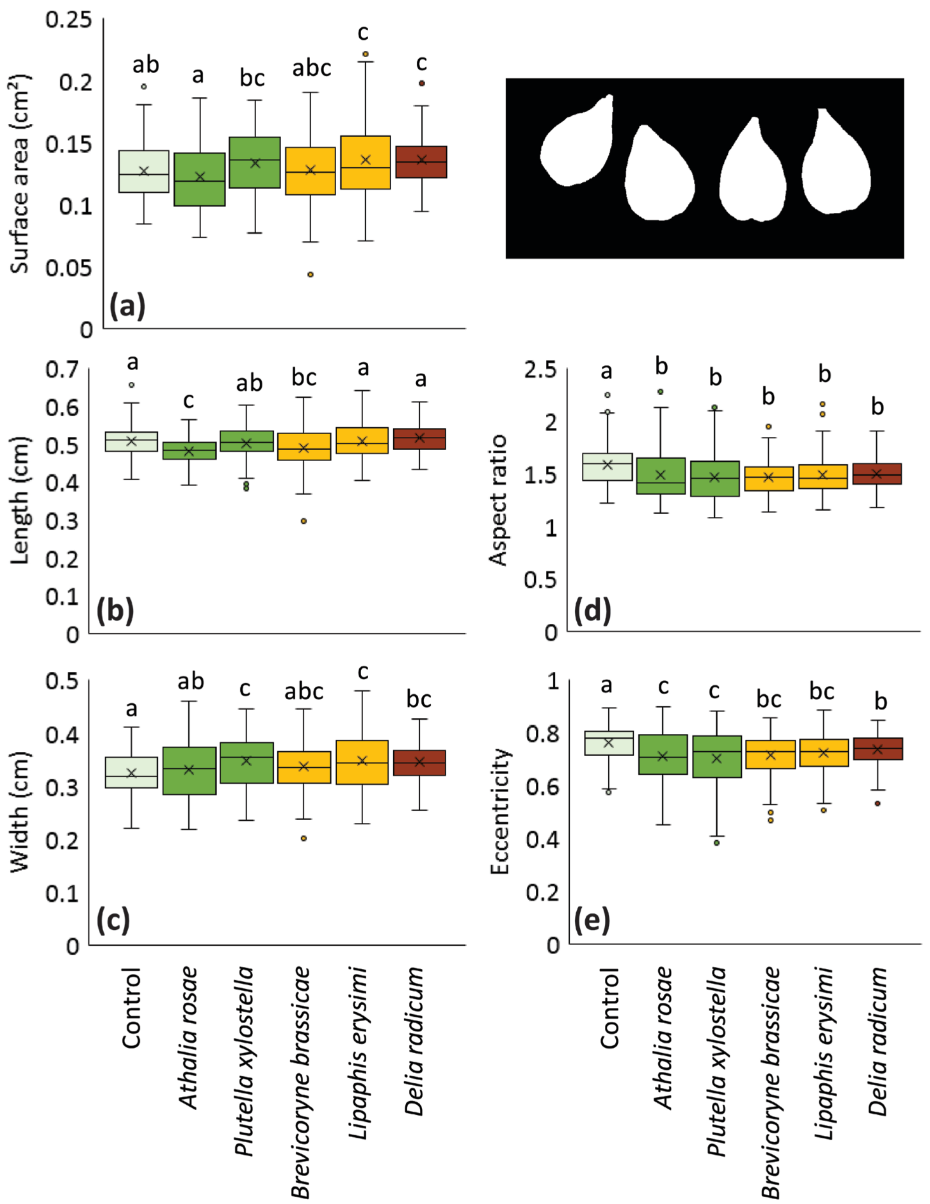

Fig. S3 Morphometry for petals of uninfested *Brassica nigra* plants and plants infested with different herbivores. We measured a) surface area, b) major chord length, c) minor chord length, d) aspect ratio, and e) eccentricity. Boxplots show median (line), mean (x), 1st and 3rd quartiles, minimum and maximum. Outliers (1.5 times the interquartile range below the 1st or above the 3rd quartile) are represented by circles. Measurements were taken after seven days of herbivory, by taking pictures of all petals from each flower and subsequent software processing. Number of replicates per herbivore treatment varied between seven and eight plants, six flowers were used from each plant. Letters above bars indicate significant differences at $\alpha = 0.05$ based on Tukey's *post hoc* tests.

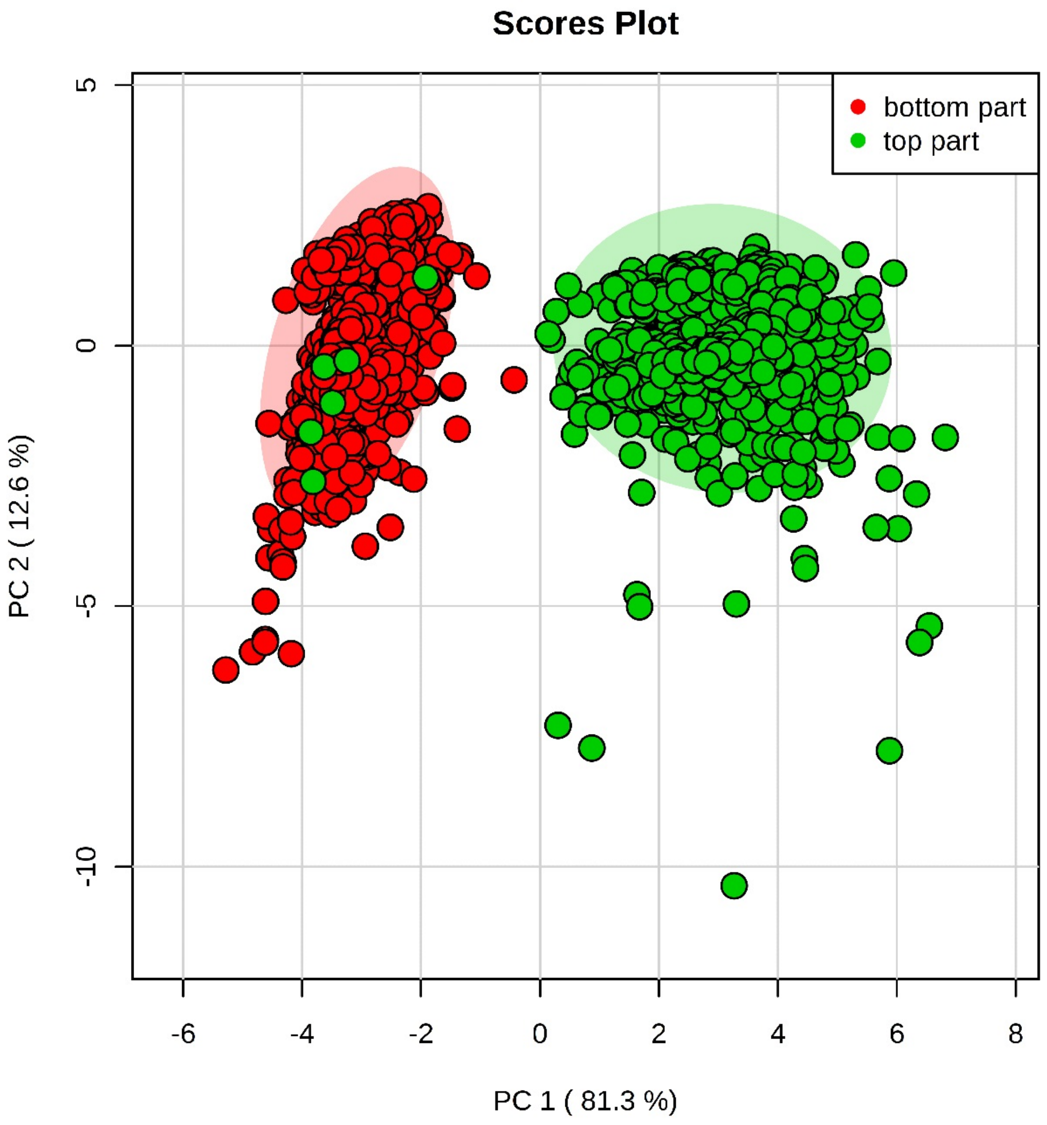

Fig. S4 Principal component analysis of the color profile of the top (green) and base (red) part of petals of *Brassica nigra* flowers. Highlighted areas denote 95% confidence intervals. Reflectance spectra measurements were done after seven days of herbivory. In total, the top and base parts of 1080 petals were measured, from 270 flowers from 45 plants.

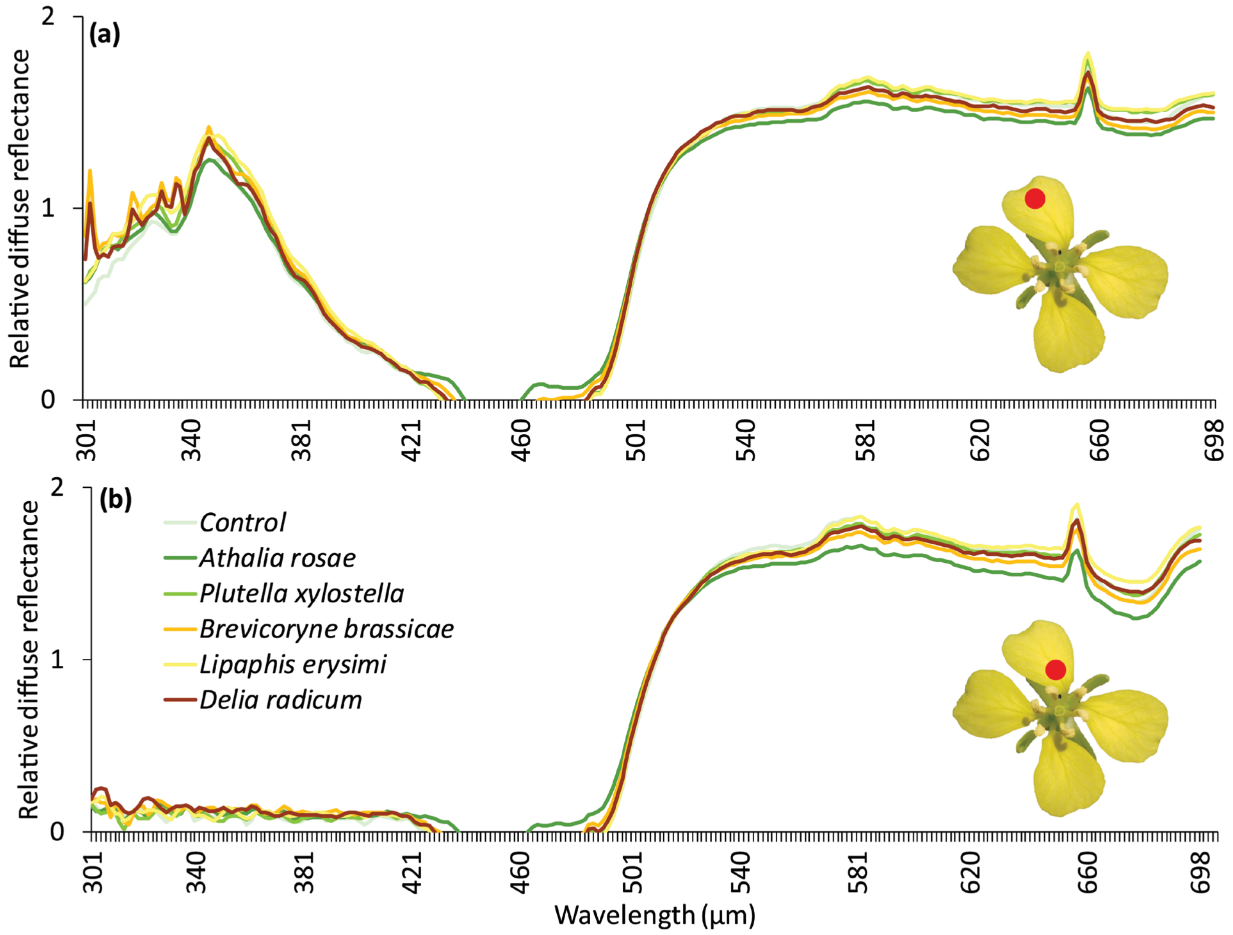

Fig. S5 Reflectance spectra with relative diffuse reflectance of wavelengths (300-700 nm) of the top (a) and base (b) part of petals of *Brassica nigra* plants infested with different herbivores or uninfested plants. The flower with the red dot indicates where measurements were taken (top or base), which was done after seven days of herbivory. Number of replicates per herbivore treatment varied between six and eight plants, and from each plant, all four petals of six flowers were measured.

Table S2. Confusion matrices of support vector machine classifiers for the reflectance spectra of top parts of petals of uninfested *Brassica nigra* plants or plants infested with different herbivores (a) or herbivore functional groups (HFGs) (b). Herbivore functional groups were assigned as followed: Chewing herbivores: *Athalia rosae* and *Plutella xylostella*; sap-feeding herbivores: *Brevicoryne brassicae* and *Lipaphis erysimi*; root herbivores: *Delia radicum*. The models assigned individual herbivore species to the correct HFGs (c) and *vice versa* (d) based on the reflectance spectra. The flower with the red dot indicates where measurements were taken (top or base), which was done after seven days of herbivory. Number of replicates per herbivore treatment varied between six and eight plants, and from each plant, all four petals of six flowers were measured.

(a)

Predicted by SVM model	True label: Control	*Athalia rosae*	*Plutella xylostella*	*Brevicoryne brassicae*	*Lipaphis erysimi*	*Delia radicum*
Control	57	1	0	0	1	0
Athalia rosae	0	41	7	0	0	0
Plutella xylostella	0	4	43	1	0	0
Brevicoryne brassicae	0	0	1	31	6	0
Lipaphis erysimi	1	0	0	8	42	0
Delia radicum	0	0	0	0	1	43

Accuracy	89 %
Error rate	10 %

(b)

Predicted by SVM model	True label: Control	Chewing herbivores	Sap-feeding herbivores	Root herbivores
Control	56	0	1	0
Chewing herbivores	1	96	1	0
Sap-feeding herbivores	1	1	87	0
Root herbivores	0	0	0	43

Accuracy	98 %
Error rate	2 %

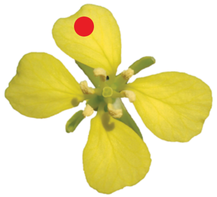

(c)

Predicted by SVM model	True label: Control	*Athalia rosae*	*Plutella xylostella*	*Brevicoryne brassicae*	*Lipaphis erysimi*	*Delia radicum*
Control	56	0	0	0	1	0
Chewing herbivores	1	46	50	1	0	0
Sap-feeding herbivores	1	0	1	39	48	0
Root herbivores	0	0	0	0	0	43

Accuracy	97 %

(d)

Predicted by SVM model	True label: Control	Chewing herbivores	Sap-feeding herbivores	Root herbivores
Control	57	1	1	0
Athalia rosae	0	48	0	0
Plutella xylostella	0	47	1	0
Brevicoryne brassicae	0	1	38	0
Lipaphis erysimi	1	0	49	0
Delia radicum	0	0	0	43

Accuracy	91 %

Table S3. Confusion matrices of support vector machine classifiers for the reflectance spectra of base parts of petals of uninfested *Brassica nigra* plants or plants infested with different herbivores (a) or herbivore functional groups (HFGs) (b). Herbivore functional groups were assigned as followed: Chewing herbivores: *Athalia rosae* and *Plutella xylostella*; sap-feeding herbivores: *Brevicoryne brassicae* and *Lipaphis erysimi*; root herbivores: *Delia radicum*. The models assigned individual herbivore species to the correct HFGs (c) and *vice versa* (d) based on the reflectance spectra. The flower with the red dot indicates where measurements were taken (top or base), which was done after seven days of herbivory. Number of replicates per herbivore treatment varied between six and eight plants, and from each plant, all four petals of six flowers were measured.

(a)

Predicted by SVM model	True label: Control	*Athalia rosae*	*Plutella xylostella*	*Brevicoryne brassicae*	*Lipaphis erysimi*	*Delia radicum*
Control	63	0	1	0	2	0
Athalia rosae	0	38	8	0	0	0
Plutella xylostella	0	8	39	0	0	0
Brevicoryne brassicae	1	0	2	31	5	0
Lipaphis erysimi	1	0	0	4	43	2
Delia radicum	0	0	0	0	0	38

Accuracy	83 %
Error rate	12 %

(b)

Predicted by SVM model	True label: Control	Chewing herbivores	Sap-feeding herbivores	Root herbivores
Control	64	0	1	0
Chewing herbivores	0	92	0	0
Sap-feeding herbivores	1	4	84	2
Root herbivores	0	0	0	38

Accuracy	92 %
Error rate	4 %

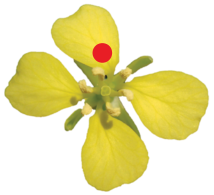

(c)

Predicted by SVM model	True label: Control	*Athalia rosae*	*Plutella xylostella*	*Brevicoryne brassicae*	*Lipaphis erysimi*	*Delia radicum*
Control	64	0	0	0	1	0
Chewing herbivores	0	46	47	0	0	0
Sap-feeding herbivores	1	0	4	35	49	2
Root herbivores	0	0	0	0	0	38

Accuracy	93 %

(d)

Predicted by SVM model	True label: Control	Chewing herbivores	Sap-feeding herbivores	Root herbivores
Control	63	1	2	0
Athalia rosae	0	46	0	0
Plutella xylostella	0	47	0	0
Brevicoryne brassicae	1	2	36	0
Lipaphis erysimi	1	0	47	2
Delia radicum	0	0	0	38

Accuracy	84 %

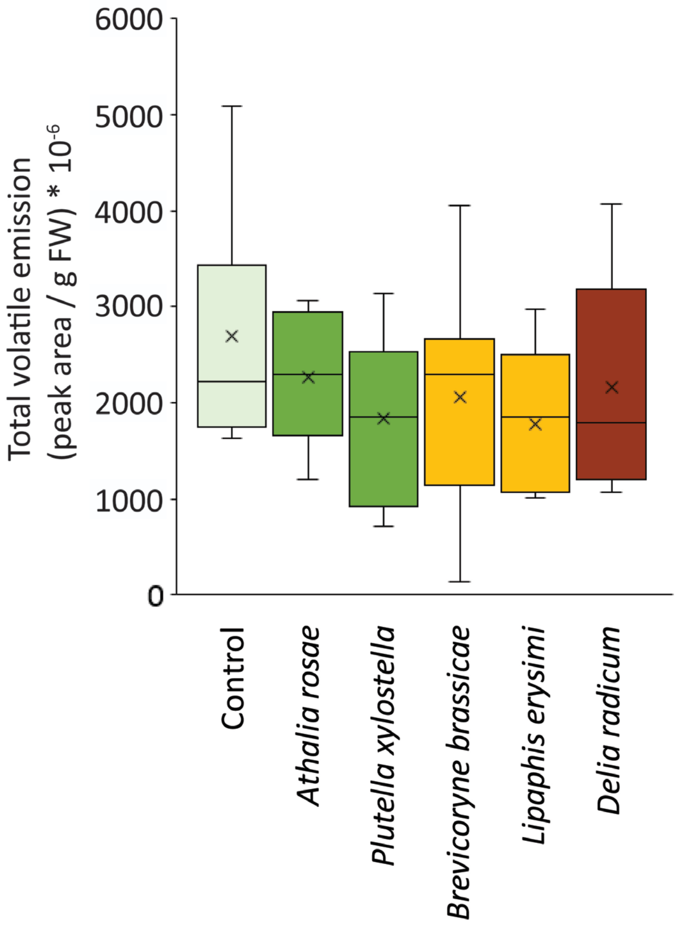

Fig. S6 Total volatile emission (peak area/ g FW) of uninfested flowering *Brassica nigra* plants and plants infested with different herbivores. Boxplots show median (line), mean (x), 1st and 3rd quartiles, minimum and maximum. Volatiles were collected after seven days of herbivory. Number of replicates per herbivore treatment varied between seven and nine plants.

Table S4. Volatile compounds of uninfested flowering *Brassica nigra* plants or plants infested with different herbivores. Volatiles were collected after seven days of herbivory. Peak area of volatile emission for each compound was corrected by g FW and divided by 10^5. Number of replicates per herbivore treatment varied between seven and nine plants.

**Putatively identified volatile compounds**	**Arithmetic Index***	Uninfested control<br>Peak area / g FW<br>Mean ± SD	*Athalia rosae*<br>Peak area / g FW<br>Mean ± SD	*Plutella xylostella*<br>Peak area / g FW<br>Mean ± SD	*Brevicoryne brassicae*<br>Peak area / g FW<br>Mean ± SD	*Lipaphis erysimi*<br>Peak area / g FW<br>Mean ± SD	*Delia radicum*<br>Peak area / g FW<br>Mean ± SD
**Benzenoids and phenylpropanoids**							
Benzaldehyde	**971**	3529 ± 2601	2181 ± 765	2334 ± 1368	1940 ± 954	2198 ± 1311	2345 ± 1032
Benzyl alcohol	**1039**	1048 ± 1228	787 ± 339	607 ± 417	615 ± 435	782 ± 532	898 ± 383
Phenylacetaldehyde	**1053**	2848 ± 3490	1829 ± 1690	1265 ± 1402	1664 ± 979	1377 ± 1402	1328 ± 1294
Benzyl acetate	**1148**	39 ± 62	23 ± 18	18 ± 17	20 ± 12	26 ± 27	28 ± 21
Methyl salicylate	**1207**	45 ± 97	10 ± 5	17 ± 29	31 ± 55	12 ± 11	15 ± 9
p-Anisaldehyde	**1269**	2227 ± 2418	1357 ± 631	1597 ± 1137	1074 ± 606	1106 ± 1099	1152 ± 734
**Monoterpenoids**							
α-Thujene	**932**	810 ± 588	662 ± 329	390 ± 290	569 ± 458	435 ± 350	568 ± 454
α-Pinene	**943**	1423 ± 556	1520 ± 373	1274 ± 827	1222 ± 618	1256 ± 454	1368 ± 409
Camphene	**960**	549 ± 622	332 ± 301	193 ± 161	429 ± 404	212 ± 188	297 ± 361
Sabinene	**981**	889 ± 428	807 ± 361	501 ± 367	643 ± 424	568 ± 330	679 ± 467
β-Pinene	**989**	286 ± 145	236 ± 132	176 ± 126	200 ± 143	172 ± 104	216 ± 172
β-Myrcene	**991**	382 ± 257	329 ± 121	193 ± 128	238 ± 159	246 ± 153	255 ± 172
α-Phellandrene	**1015**	212 ± 147	178 ± 94	95 ± 76	145 ± 119	118 ± 100	146 ± 114
α-Terpinene	**1025**	119 ± 173	64 ± 56	36 ± 38	67 ± 75	35 ± 31	59 ± 68
p-Cymene	**1033**	2 ± 2	1 ± 1	1 ± 1	1 ± 1	1 ± 1	1 ± 1
β-Ocimene, (*E*)-	**1050**	979 ± 566	623 ± 630	323 ± 430	708 ± 632	409 ± 562	641 ± 731
γ-Terpinene	**1065**	66 ± 72	45 ± 33	26 ± 20	41 ± 38	28 ± 22	39 ± 39
Terpinolene	**1094**	276 ± 279	195 ± 127	102 ± 87	168 ± 150	120 ± 89	168 ± 156
α-Pinene oxide	**1115**	21 ± 20	27 ± 28	9 ± 9	25 ± 35	20 ± 24	12 ± 9
Alloocimene, neo	**1131**	1319 ± 603	1061 ± 490	839 ± 610	935 ± 678	774 ± 415	1028 ± 678
2,6-Dimethyl-1,3,5,7-octatetraene, (*Z*)-	**1136**	53 ± 39	30 ± 29	32 ± 45	40 ± 36	22 ± 31	34 ± 34
β-Ocimene epoxide, (*E*)-	**1142**	32 ± 11	14 ± 9	22 ± 18	42 ± 59	17 ± 12	27 ± 22
Verbenol, (*E*)	**1158**	57 ± 27	38 ± 29	22 ± 25	40 ± 35	21 ± 18	26 ± 15
Pinocarvone	**1178**	76 ± 67	51 ± 37	35 ± 41	54 ± 43	32 ± 24	39 ± 37
α-Terpineol	**1207**	143 ± 122	87 ± 58	61 ± 56	64 ± 43	57 ± 35	78 ± 51
Myrtenal	**1211**	96 ± 84	64 ± 42	44 ± 49	66 ± 53	39 ± 26	52 ± 48
Verbenone	**1225**	514 ± 253	540 ± 231	203 ± 182	294 ± 218	402 ± 387	532 ± 326

Putatively identified volatile compounds	Arithmetic Index*	Uninfested control<br>Peak area / g FW<br>Mean ± SD	*Athalia rosae*<br>Peak area / g FW<br>Mean ± SD	*Plutella xylostella*<br>Peak area / g FW<br>Mean ± SD	*Brevicoryne brassicae*<br>Peak area / g FW<br>Mean ± SD	*Lipaphis erysimi*<br>Peak area / g FW<br>Mean ± SD	*Delia radicum*<br>Peak area / g FW<br>Mean ± SD
**Homoterpenoids**							
4,8-Dimethyl-1,3,7-nonatriene, (*E*)-	**1115**	303 ± 310	458 ± 243	435 ± 459	266 ± 122	197 ± 133	518 ± 675
4,8,12-Trimethyltrideca-1,3,7,11-tetraene , (*E, E*)-	**1578**	34 ± 17	93 ± 118	174 ± 209	241 ± 307	61 ± 61	40 ± 41
**Sesquiterpenoids**							
7-α-H-Silphiperfol-5-ene	**1347**	52 ± 58	48 ± 67	103 ± 133	51 ± 78	30 ± 16	117 ± 209
Presilphiperfol-7-ene	**1355**	2 ± 2	4 ± 6	4 ± 5	3 ± 5	3 ± 4	3 ± 4
7-β-H-Silphiperfol-5-ene	**1367**	14 ± 16	14 ± 21	38 ± 49	10 ± 12	9 ± 5	80 ± 190
Silphiperfol-6-ene	**1371**	8 ± 9	8 ± 12	21 ± 28	6 ± 7	5 ± 3	48 ± 116
Silphiperfol-5,7(14)-diene	**1378**	1 ± 1	1 ± 1	3 ± 5	1 ± 2	1 ± 0.3	7 ± 17
β-Caryophyllene	**1446**	3 ± 1	11 ± 12	12 ± 16	5 ± 8	3 ± 1	19 ± 39
α-Farnesene, (*Z,E*)-	**1493**	91 ± 40	79 ± 71	129 ± 194	71 ± 71	52 ± 50	73 ± 40
α-Farnesene, (*E,E*)-	**1508**	134 ± 107	131 ± 120	68 ± 58	112 ± 89	67 ± 90	108 ± 100
**Fatty-acid and/or amino-acid derivatives**							
3-Hexen-1-ol, acetate, (*Z*)-	**1004**	385 ± 251	554 ± 235	410 ± 355	363 ± 313	667 ± 494	366 ± 288
2-Ethylacetate	**1147**	107 ± 78	71 ± 35	58 ± 39	88 ± 83	53 ± 29	82 ± 83
2-Methylbutanoic acid methyl ester**		29 ± 12	26 ± 10	28 ± 29	28 ± 18	23 ± 15	37 ± 44
3-Hydroxy-2-butanone**		1 ± 1	1 ± 0.4	10 ± 27	0.4 ± 0.2	1 ± 2	2 ± 2
Tiglic aldehyde**		270 ± 197	230 ± 154	240 ± 240	349 ± 298	195 ± 77	373 ± 526
**Nitrogen and/or sulphur containing compounds**							
unknown thiocyanate	**871**	158 ± 124	136 ± 102	252 ± 270	244 ± 251	175 ± 72	146 ± 123
Allyl isothiocyanate	**885**	1594 ± 830	1440 ± 826	1973 ± 1396	1612 ± 1204	1571 ± 248	1488 ± 873
Benzyl cyanide	**1147**	1071 ± 1048	503 ± 509	397 ± 625	739 ± 595	364 ± 371	653 ± 687
Benzaldehyde, 2-amino	**1235**	1498 ± 1881	597 ± 407	581 ± 499	974 ± 761	579 ± 522	809 ± 356
Methyl thiocyanate**		4 ± 3	3 ± 2	5 ± 4	5 ± 3	3 ± 2	5 ± 4
unknown nitrile m/z 67**		1180 ± 798	1080 ± 440	1165 ± 1022	1307 ± 946	881 ± 297	1008 ± 430
**Unknown compounds**							
unkonwn m/z 134.18	**1107**	35 ± 29	25 ± 16	17 ± 14	21 ± 15	17 ± 10	23 ± 19
unknown m/z 108.14	**1138**	149 ± 87	154 ± 139	66 ± 75	127 ± 161	114 ± 94	63 ± 38
unknown m/z 150.17	**1429**	28 ± 20	16 ± 17	9 ± 11	24 ± 23	12 ± 16	19 ± 20

* Calculation of Arithmetic Index (AI) as described by Adams (2001) Identification of Essential Oil Components by Gas Chromatography/Mass Spectrometry. USA: Allured Books.
** We did not calculate the arithmetic index for this compound because the shortest chain linear hydrocarbon we injected was octane

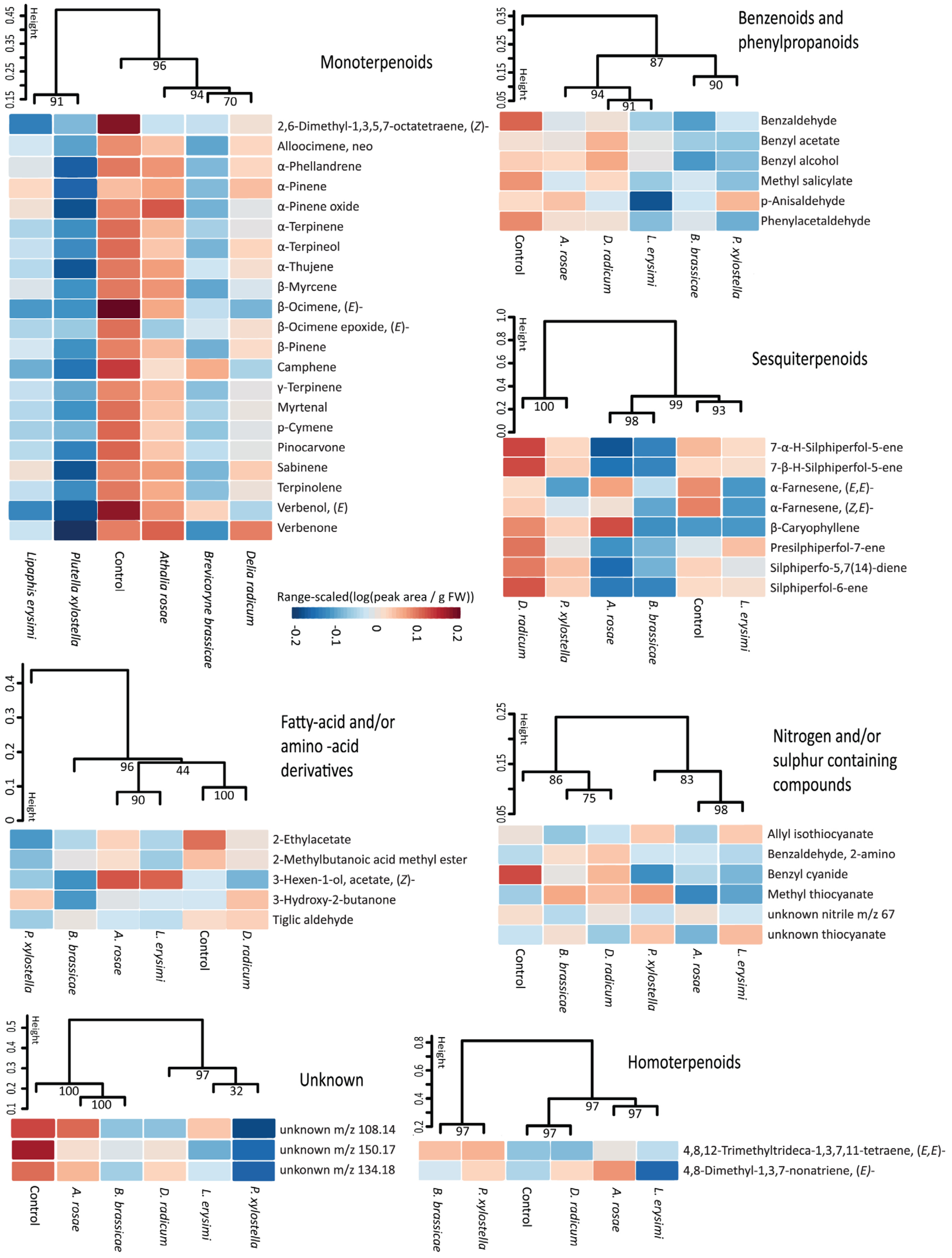

Fig. S7 Dendrogram and heat map of the emission of volatile compounds for each compound class of *Brassica nigra* plants infested with different herbivores or uninfested plants. Dendrogram clustering was performed using Ward's clustering algorithm with Euclidean distances. Values in the dendrogram are approximately unbiased probability values. For the heat map, we used range-scaled log transformed values of volatile emission (peak area / g FW) for each compound. Volatiles were collected after seven days of herbivory. Number of replicates per herbivore treatment varied between seven and nine plants.

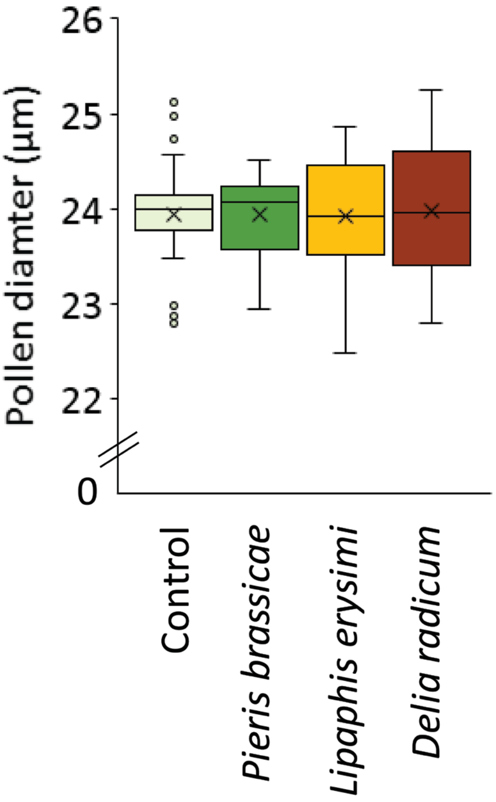

Fig. S8 Size of pollen grains of uninfested *Brassica nigra* plants or plants infested with different herbivores. Boxplots show median (line), mean (x), 1st and 3rd quartiles, minimum and maximum. Outliers (1.5 times the interquartile range below the 1st or above the 3rd quartile) are represented by circles. Measurements were done after seven days of herbivory. Number of replicates per herbivore treatment was 10 plants, per plant we measured pollen for five flowers.

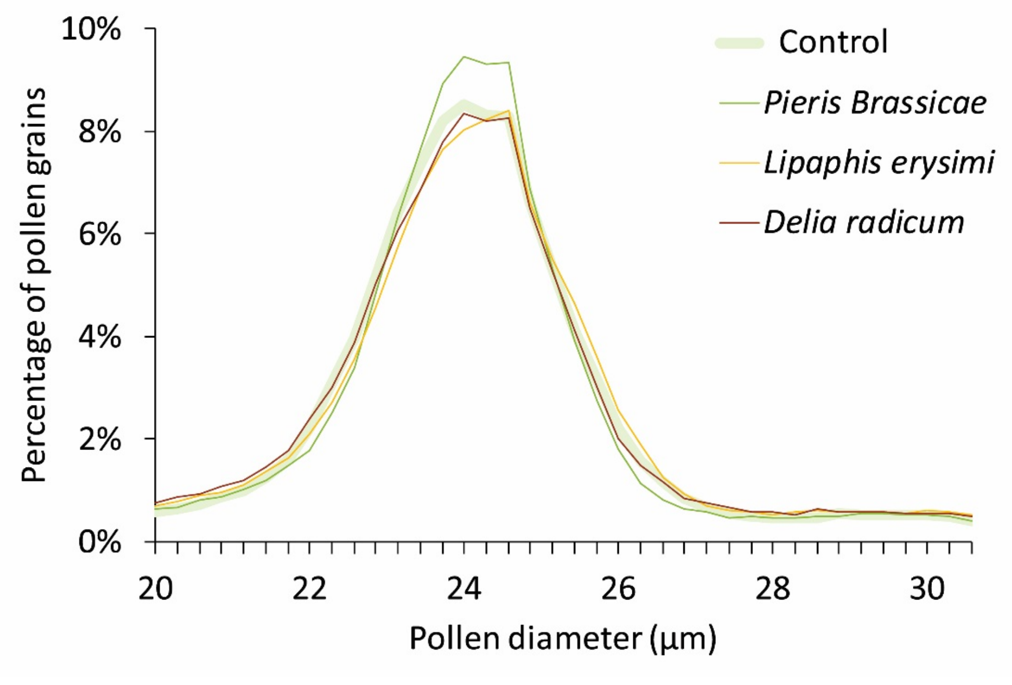

Fig. S9 Size distribution of pollen grains of uninfested *Brassica nigra* plants or plants infested with different herbivores. Measurements were done after seven days of herbivory. Number of replicates per herbivore treatment was 10 plants, per plant we measured pollen for five flowers.

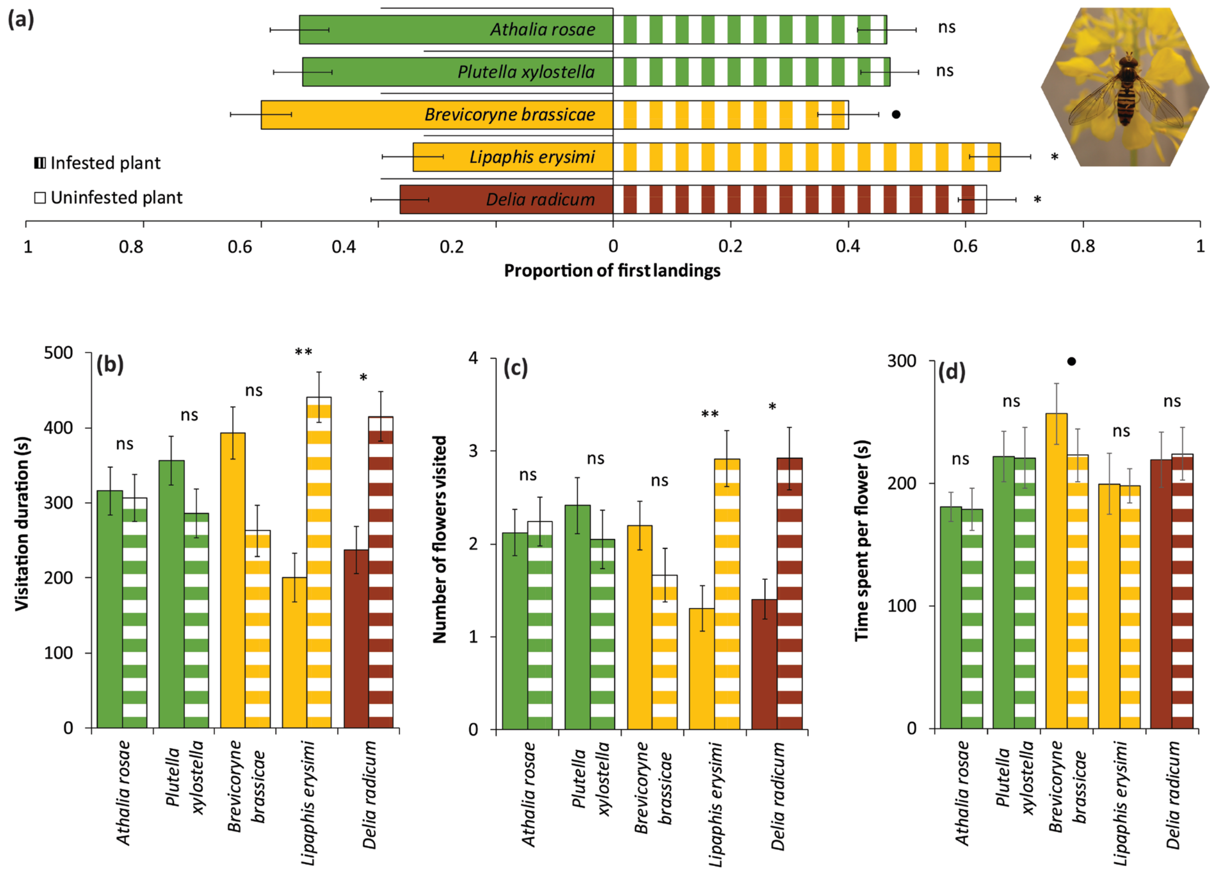

Fig. S10 Preference of the syrphid fly *Episyrphus balteatus* for uninfested *Brassica nigra* plants or plants infested with different herbivores. (a) Proportion of *E. balteatus* syrphid flies (mean ± SE) that first landed on flowers or leaves of *Brassica nigra* plants infested with different herbivores or uninfested plants. b) Visitation duration (mean ± SE); c) number of flowers visited (mean ± SE); and d) time spent per flower (mean ± SE) by individual pollinators on infested or uninfested *B. nigra* plants. Syrphid fly behavior was assessed after seven days of herbivory. Number of replicates per herbivore treatment varied between 85 and 102 syrphid flies, and 9 and 11 plant pairs. Asterisks above bars indicate significant differences with *** = $P < 0.001$, ** = $0.001 \geq P < 0.01$, * = $0.01 \geq P \leq 0.05$, and ● = $0.05 > P < 0.1$, based on Tukey's *post hoc* tests. Photograph shows an *E. balteatus* syrphid fly visiting flowers of *B. nigra*. Photograph credit: Quint Rusman.
